# Immobilization of Biantennary N-Glycans Leads to Branch Specific Epitope Recognition by LSECtin

**DOI:** 10.1021/acscentsci.2c00719

**Published:** 2022-09-20

**Authors:** Sara Bertuzzi, Francesca Peccati, Sonia Serna, Raik Artschwager, Simona Notova, Michel Thépaut, Gonzalo Jiménez-Osés, Franck Fieschi, Niels C. Reichardt, Jesús Jiménez-Barbero, Ana Ardá

**Affiliations:** † Basque Research & Technology Alliance (BRTA), Chemical Glycobiology Group, CIC bioGUNE, Bizkaia Technology Park, Building 800, 48160 Derio, Bizkaia, Spain; 9 Basque Research & Technology Alliance (BRTA), Computational Chemistry Group, CIC bioGUNE, Bizkaia Technology Park, Building 800, 48160 Derio, Bizkaia, Spain; ‡ Glycotechnology Group, Basque Research and Technology Alliance (BRTA), CIC biomaGUNE, Paseo Miramón 182, 20014 San Sebastian, Spain; § Memorial Sloan Kettering Cancer Center, 417 East 68th Street, New York, New York 10065, United States; ∥ CNRS, CEA, Institut de Biologie Structurale, University of Grenoble Alpes, 38000 Grenoble, France; ⊥ Ikerbasque, Basque Foundation for Science, Maria Diaz de Haro 3, 48013 Bilbao, Bizkaia, Spain; # CIBER-BBN, Paseo Miramón 182, 20009 San Sebastian, Spain; ∇ Department of Organic Chemistry, II Faculty of Science and Technology University of the Basque Country, EHU-UPV, 48940 Leioa, Spain; ○ Centro de Investigación Biomédica En Red de Enfermedades Respiratorias, 28029 Madrid, Spain

## Abstract

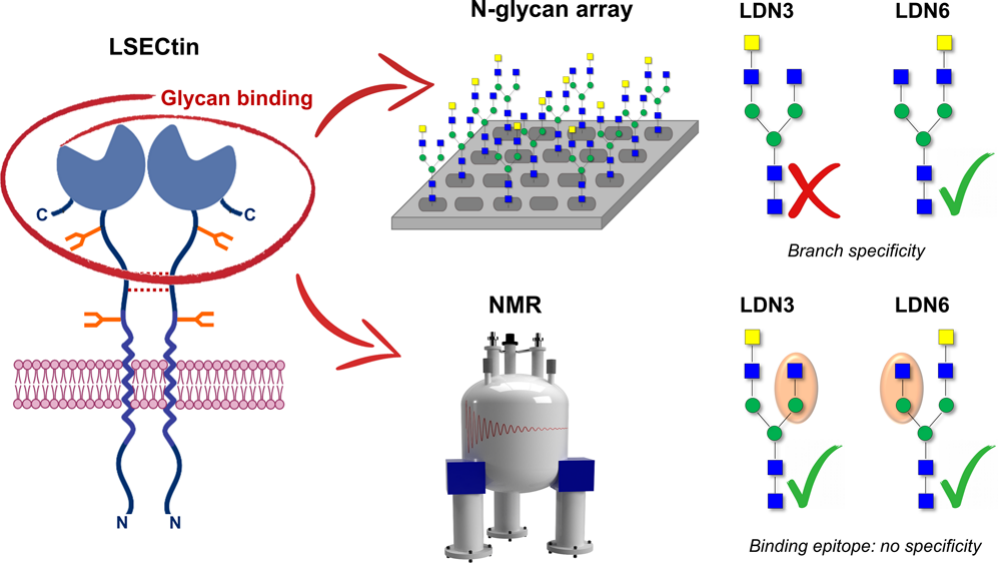

The molecular recognition features of LSECtin toward asymmetric N-glycans have been scrutinized by NMR and compared to those occurring in glycan microarrays. A pair of positional glycan isomers (LDN3 and LDN6), a nonelongated GlcNAc4Man3 N-glycan (G0), and the minimum binding epitope (the GlcNAcβ1-2Man disaccharide) have been used to shed light on the preferred binding modes under both experimental conditions. Strikingly, both asymmetric LDN3 and LDN6 N-glycans are recognized by LSECtin with similar affinities in solution, in sharp contrast to the results obtained when those glycans are presented on microarrays, where only LDN6 was efficiently recognized by the lectin. Thus, different results can be obtained using different experimental approaches, pointing out the tremendous difficulty of translating *in vitro* results to the *in vivo* environment.

## Abstract 2

Solution NMR and surface-based microarray studies provide different results on the molecular recognition features of LSECtin toward diverse N-glycans. Which one is closer to those existing in nature?

## Introduction

The molecular recognition of glycans by specialized glycan binding proteins, lectins, is at the heart of the constant struggle between hosts and pathogens.^1^ Humans possess an army of lectins in charge of recognizing exogenous glycans^2^ with different roles in the infectious process. C-type lectin receptors^3^ are included in a subfamily of pattern recognition receptors (PPRs) in charge of sensing specific glycan signatures on pathogens.^4^ These lectins are nowadays recognized as crucial modulators of the delicate balance between disease and homeostasis, where besides binding to glycans on the pathogen surface, they are also involved in cell adhesion and endocytic processes through binding to self-glycoproteins.^5^

Our knowledge about the molecular basis of the interactions between lectins and glycans has grown in the last few decades,^6^ fostered by diverse scientific and technological advances.^7^ However, our understanding of the rules governing these associations is not yet complete. A particular feature of glycan–protein interactions is the generally weak affinity toward short oligosaccharides, which can be compensated by multivalency, both from the glycan and receptor sides.^8^ Additionally, glycan presentation, which is related to different effects such as orientation, dynamics, flexibility, accessibility, and density, is also known to be determinant.^9^ As a result, the design of molecules able to effectively modulate glycan–lectin interactions in the natural context remains elusive.

Among C-type lectins, LSECtin (CLEC4G) is a transmembrane receptor described for the first time in 2004, a member of the C-type lectin family of Ca^2+^-dependent glycan binding receptors.^10^ It was initially found to be expressed in liver, lymph nodes, bone marrow, and sinusoidal endothelial cells^11^ and subsequently was also detected in liver Kupffer cells and isolated *ex vivo* from human peripheral blood and thymic dendritic cells.^12,13^ Although its 3D structure is not yet available, sequence analysis has revealed a close relationship with the C-type lectins DC-SIGN and DC-SIGNR, all being encoded in the same gene cluster 19p13.2.^14^

**Figure 1:**
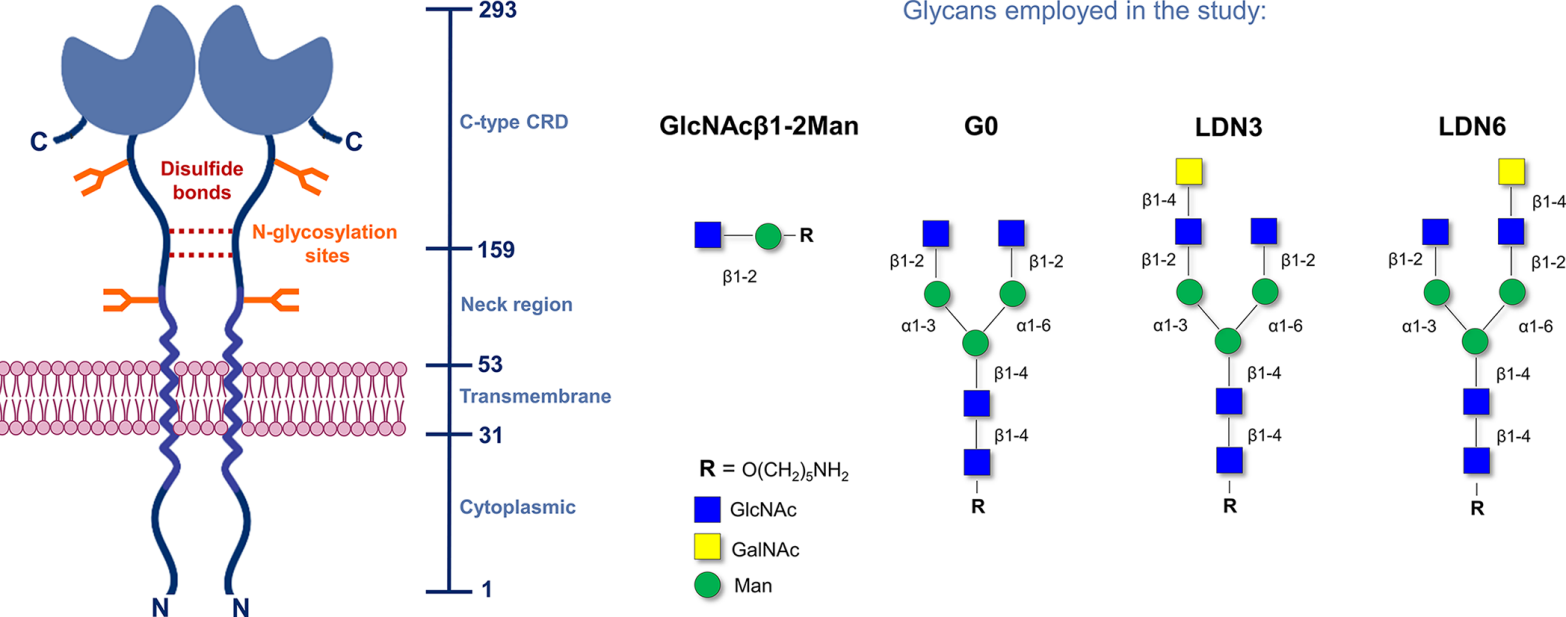
LSECtin model and ligands employed in this study. On the left-hand side: model of the structure of the full LSECtin dimer. The sequence of the monomer contains 293 amino acids with a total molecular weight of 32 kDa. On the right-hand side: the glycans used herein with their respective nomenclature. In the NMR study, the CRD has been used.

The general structure of the full-length receptor is composed of an extracellular domain (ECD), a transmembrane hydrophobic domain, and an intracellular N-terminal tail of 31 amino acids. The ECD can be further divided (Figure 1) into a neck region (110 amino acids) and a carbohydrate recognition domain (CRD) of 129 residues.^15^ Two N-glycosylation sites are present at positions 77 and 159 of the neck region, and both have been shown to be essential for efficient expression of LSECtin on the cell surface.^16^ Cysteine residues in the neck region are responsible for the dimerization of the receptor through disulfide bonds.^15^

LSECtin is a receptor of the innate immune system with major biological functions in the recognition and internalization of both self-and nonself glycoconjugates.^11,12,16^ It is a mediator of cell adhesion, glycoprotein clearance, and signaling events and is also implicated in pathogen invasion.^14^ LSECtin has been reported as an attachment factor for Ebola virus, which is likely to attach to the lectin via the terminal GlcNAc residue of viral surface N-glycans.^15,17^ LSECtin also interacts with the spike protein of the severe acute respiratory syndrome (SARS) coronavirus, thus facilitating virus infection. This interaction seems to be glycan-mediated but does not involve Man residues on the virus surface since it is inhibited by the chelating agent EGTA but not by a mannan.^16^

More recently, SARS-CoV-2 has also been shown to bind to LSECtin, an interaction proposed to be on cargo of a specific GlcNAc terminating N-glycosylation site and interfering with the ACE2/spike interaction.^18^ Moreover, the presence of LSECtin enhances the binding of Lassa virus to host cells and enhances the infection of lymphocytic choriomeningitis virus (LCMV).^19,20^

LSECtin has also been found to be overexpressed in tumor-associated macrophages in breast cancer.^21^ With respect to the glycan binding preferences, it has been reported that LSECtin is able to recognize Man, Fuc, Glc, and GlcNAc monosaccharides in a calcium-dependent manner with a slight preference for Man and GlcNAc moieties.^10^ Glycan array studies outline a preference for the GlcNAcβ1-2Man moiety of terminal N-linked glycoproteins with an experimental dissociation constant (*K*_d_) for the disaccharide unit of 3.5 μM, which is a surprisingly strong affinity when comparing disaccharide binding to DC-SIGN and DC-SIGNR.^15^

A recent study conducted in our laboratory employing glycan arrays, highlighted a clear preference of this lectin for the GlcNAcβ1-2Man motif in asymmetrical N-glycans decorated at the Manα1-6 arm over the positional isomers at the Manα1-3 arm. It was shown that complex and hybrid type glycans with a terminating GlcNAcβ1-2Man moiety at the 3-branch were capable of binding to LSECtin, independently of the glycan composition at the 6-branch. In particular, complex type N-glycans bearing GalNAc/Galβ1-4 elongations at the 6-arm were recognized by LSECtin, while the same decoration at the 3-arm completely abolished the binding event.^22^ This reported exquisite preference and selectivity provide an example of how a subtle difference in the glycan structure may lead to significant changes in the binding event^23−28^ and, moreover, can be considered as a key starting point to be exploited to achieve selective inhibition of the lectin.

Nevertheless, although high-throughput glycan array analysis is a powerful tool for an initial ligand screening,^29,30^ we considered that additional investigations were needed to further validate and understand the observations using arrays with other experimental conditions and protocols.

## Results

### The Interaction of LSECtin with the Minimum Epitope GlcNAcβ1-2Man by NMR

The NMR study started by examining the interaction between the minimum binding epitope, the GlcNAcβ1-2Man disaccharide, and LSECtin, for which a binding affinity of 3.5 μM has been reported using a solid phase binding competition assay.^15^ For the binding analysis, the monomeric, soluble CRD portion of LSECtin was employed.

**Figure 2:**
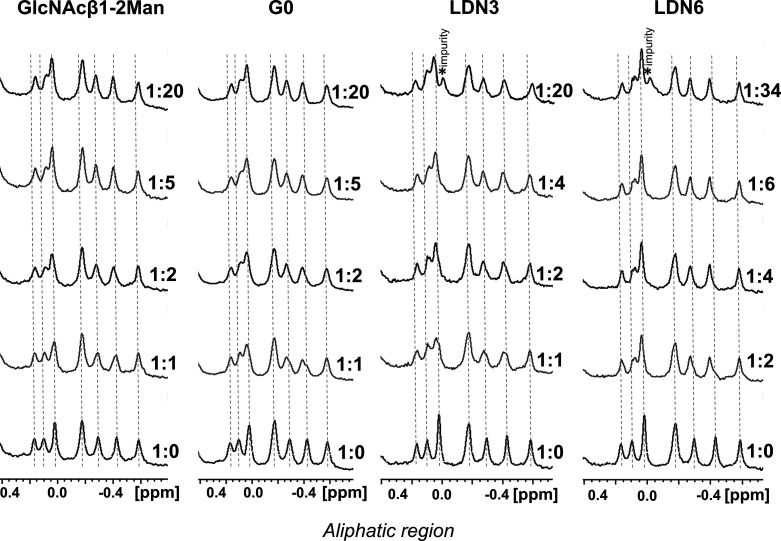
Stacked ^1^H NMR spectra acquired during the titration of (from the left) GlcNAcβ1-2Man, G0, LDN3, and LDN6 to LSECtin. Amounts of equivalents are reported for each spectrum. Expansion of the high field region (aliphatic protons) of the spectra is shown.

A ^1^H NMR-based titration experiment was carried out by adding increasing amounts of the ligand (120 μM, 240 μM, 600 μM, 1.2 mM, 2.4 mM) to a sample containing the lectin (120 μM in deuterated buffer), resulting in protein/ligand ratios of 1:0 (apo lectin), 1:1, 1:2, 1:5, 1:10, and 1:20. Perturbations on the protein ^1^H NMR signals upon ligand addition were monitored at each titration point. From the superimposed ^1^H NMR spectra acquired during the titration (Figure 2 left and Figures S3, S8, and S12 in the Supporting Information) clear changes for some NMR signals were evident upon addition of 1 and 2 equivalents of ligand. Protein saturation was reached after the addition of 2 equivalents of ligand and no further changes in the protein spectrum were observed at higher lectin:ligand ratios. For low ligand/protein ratios, the system displayed slow exchange in the chemical shift time scale between the free and bound protein signals. These observations are in good agreement with the reported low micromolar affinity for this interaction.

With the aim of better understanding the binding features of the system, saturation transfer difference NMR (STD-NMR) experiments^31−33^ were employed since they constitute a powerful ligand-based NMR tool to study protein–ligand interactions, including the assessment of the ligand binding epitope. Different experimental conditions were screened, with the best results (signal-to-noise ratio) being obtained at 310 K, with a lectin:ligand molar ratio of 1:70. The STD-NMR spectrum, under aliphatic irradiation (Figure S4), showed clear STD signals for several protons that could be unambiguously identified, namely H2, H6, H6′and H4/H5 (overlapped) of the GlcNAc residue, as well as H3 of the Man residue. The methyl group signal from the N-acetyl group (NAc) showed relatively weaker STD intensity. Additionally, a ROESY-NMR experiment was acquired at a 1:10 lectin/ligand ratio, which showed chemical exchange cross-peaks for a number of ligand protons, indicating that the process is in the slow exchange regime on the chemical shift time scale (Figure S5). This circumstance allowed to reveal the chemical shifts of the ligand signals in the bound state, that is, the chemical shift perturbations undergone upon protein binding, thus providing additional information on the interacting ligand epitope.^34^ In this case, most protons of the GlcNAc ring suffered a significant upfield shift, being especially remarkable for H6′and H4, strongly suggesting that these protons (both displaying axial-like orientation on the β-pyranose ring face) are in front of an aromatic ring of the lectin in the complex.^34,35^ Interestingly, H1, H2, and H3 of the Man residue experience the opposite effect, shifting downfield, confirming that the Man residue is also importantly associated with the lectin in the bound complex but with a completely different chemical environment to that of GlcNAc (Figure S5).

### The Interaction of LSECtin with N-Glycans by NMR

Once the basic binding epitope had been defined, the interaction between LSECtin and the complex N-glycans shown in Figure 1 was studied. Specifically, the nonelongated N-glycan (G0) and the two positional isomers (LDN3 and LDN6) with an additional GalNAc residue either at the 3- or 6-arm, respectively, were employed for the molecular recognition studies. A binding affinity to LSECtin of 2.6 μM has been reported for the N-glycan G0, using solid phase binding competition assays.^15^

For the NMR study, the same experimental NMR protocol described above was carried out. Thus, increasing amounts of G0 (120 μM, 240 μM, 600 μM, 1.2 mM, 2.4 mM) were added to a sample containing the lectin (120 μM in deuterated buffer). At each titration point, a 1D ^1^H NMR spectrum was recorded, and the perturbation of the lectin NMR signals were monitored at specific protein/ligand molar ratios (1:0, 1:1, 1:2, 1:5, 1:10, and 1:20). Figure S8 in the Supporting Information, which gathers the individual NMR spectra, and Figure 2, show that the protein signals upon G0 addition display an analogous trend to that described above for the disaccharide, suggesting that the relative affinities of both molecules (G0 and GlcNAcβ1-2Man) are fairly similar. The lectin’s NMR signals show clear differences between the 1:1 and 1:2 molar ratios (see expansion of Figure S8 in the Supporting Information and Figure 2). In contrast, no changes were observed above 1:5 molar ratios. Again, the system reaches saturation at a 1:2 molar ratio.

The same NMR strategy was also employed to evaluate the interaction of LSECtin with the two positional isomers, LDN3 and LDN6, which present an additional GalNAc residue either at the 3- or at the 6-arm, respectively. Figure 2 and Figure S12 in the Supporting Information show the corresponding ^1^H NMR titrations. Interestingly, both sets of experiments share the same pattern, strongly suggesting a similar interaction with the lectin for both ligands in solution. The analysis of the spectra confirmed that protein saturation was again reached upon addition of 2–4 ligand equivalents. The comparison of the observed changes in the protein NMR signals during the titrations with the different N-glycans allowed assessing that the experimental perturbations were extremely similar among them (Figure 2) and also identical to those measured for the disaccharide.

Strikingly, no recognition preferences of LSECtin for any asymmetrical glycan was deduced from this NMR analysis in solution, in sharp contrast to the observations for the same N-glycans when immobilized on a glycan microarray.^22^

**Figure 3:**
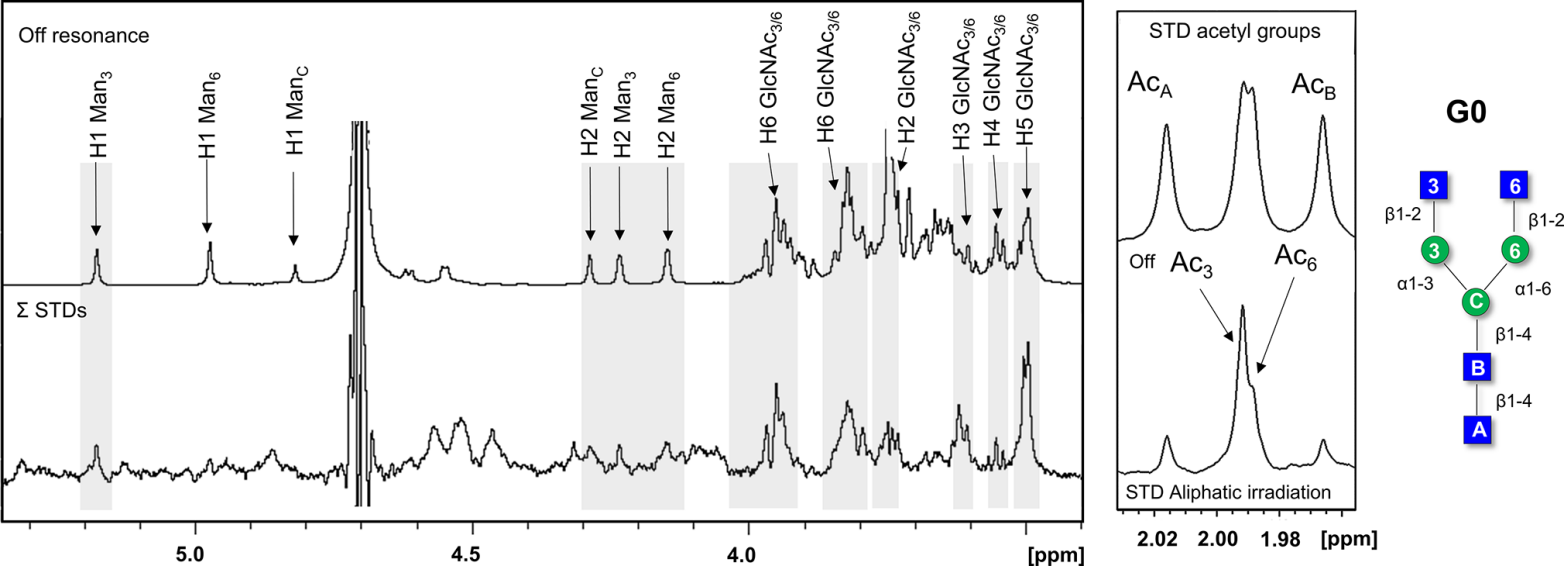
STD NMR experiments performed for the complex formed by LSECtin and G0. Left: off-resonance spectrum (irradiation at δ 100 ppm) and the sum (Σ) of STD NMR experiments carried out under aliphatic and aromatic irradiations (δ 0.6 ppm and δ 6.82 ppm, respectively). The main ^1^H NMR signals are annotated in the off-resonance spectrum. The lectin/ligand molar ratio was 1:34, with a LSECtin CRD concentration of 120 μM. The STD NMR experiments were acquired with 2 s of saturation time, 15 s of relaxation delay, and 2880 scans at 310 K. Right: expansion of the STD NMR spectra showing the acetyl group region of the GlcNAc moieties (only irradiation at the aliphatic region).

**Figure 4:**
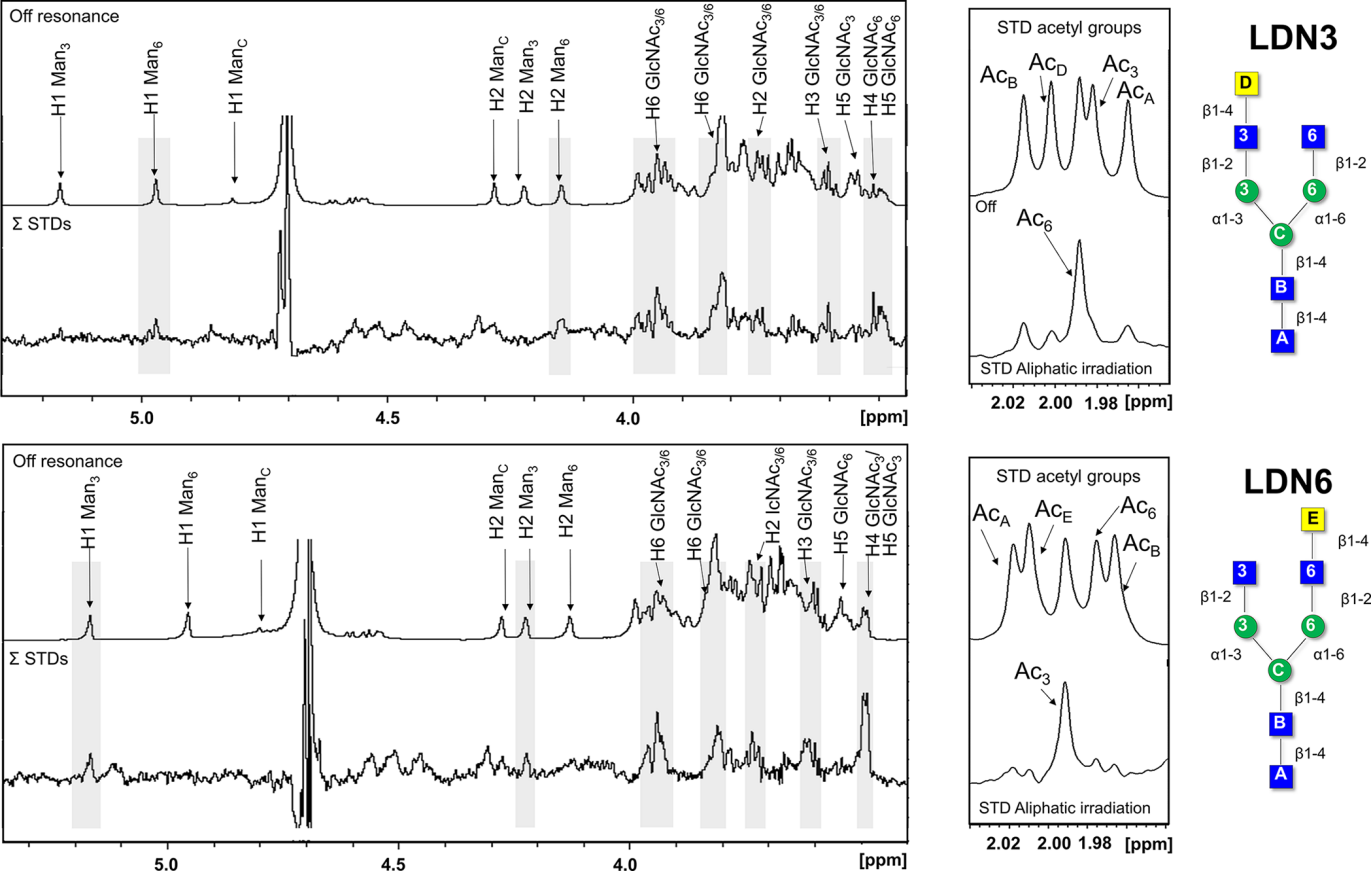
STD NMR experiments performed for the complexes formed by LSECtin and the asymmetric N-glycans (top: LDN3; bottom: LND6). Left: off-resonance spectrum (irradiation at δ 100 ppm) and the sum (Σ) of STD NMR experiments carried out under aliphatic and aromatic irradiations (δ 0.6 ppm and δ 6.82 ppm, respectively). The main ^1^H NMR signals are annotated in the off-resonance spectrum. The lectin/ligand molar ratio was 1:34, with a LSECtin CRD concentration of 120 μM. The STD NMR experiments were acquired with 2 s of saturation time, 15 s of relaxation delay, and 2880 scans at 310 K. Right: expansion of the STD NMR spectra showing the acetyl group regions of the GlcNAc moieties (only irradiation at the aliphatic region) for both glycans.

**Figure 5:**
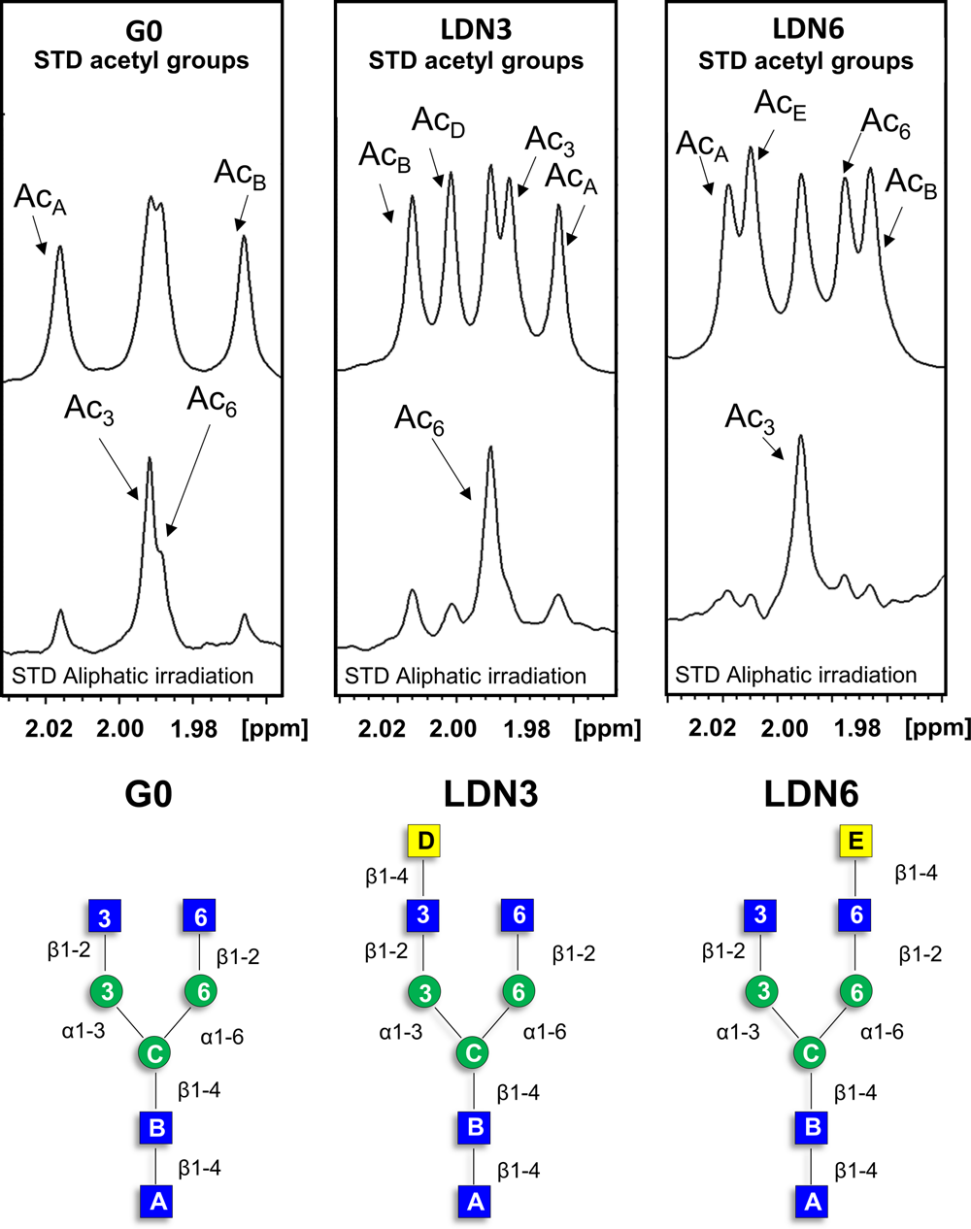
Acetyl region of the GlcNAc residues in the STD NMR spectra (bottom). The corresponding off-resonance NMR spectra (top). From left to right: the complexes of LSECtin with G0, LDN3, and LDN6, respectively. The experimental details are in the captions of Figures 3 and 4.

Additional information on the individual binding epitopes for each glycan (G0, LDN3, and LDN6) was deduced by performing STD-NMR experiments using 1:34 lectin/ligand molar ratios (Figures 3–5) and irradiation of the protein signals at the aliphatic and aromatic regions in separate experiments.

Interestingly, the STD-NMR signals for the acetyl groups were always remarkably stronger under aliphatic irradiation, suggesting that the methyl groups are close to aliphatic residues of the protein. For the nonelongated glycan G0, most of the STD-NMR signals corresponded to the two terminal GlcNAc residues, for which all protons of the pyranose ring are NMR equivalent and thus indistinguishable. The STD response was almost identical to that observed for the disaccharide indicating that this N-glycan interacts with LSECtin through the same epitope as the disaccharide. Fittingly, the ^1^H NMR signals of the acetyl groups of the two terminal GlcNAc residues in G0 display slightly different chemical shifts and could be unambiguously assigned. In particular, NOE correlations were detected between them and the anomeric proton of the Man residue at either the 3- or 6-branches (Figure S7 in the Supporting Information). The N-acetyl group signals of both terminal GlcNAc moieties of G0 displayed significant intensities in the STD NMR spectra, while those from the core GlcNAc residues (A and B) did not show any STD effect. Moreover, the STD NMR intensity observed for the Ac-GlcNAc at the 3-branch was twice as intense as for the 6-branch. Fittingly, a weak STD NMR intensity was also observed for H1 and H2 of Man3 but not for the Man-6 residue (Figure 3).

Moving to the asymmetric glycans, for LDN3, clear STD NMR signals for the 6-Man residue (H1, H2) were evident, while the same protons on the 3-Man residue did not appear in the STD NMR spectrum (Figure 4, top panel). Alternatively, for LDN6, STD NMR signals for the 3-Man residue (H1, H2) were observed, but not for the same protons of the 6-Man residue. (Figure 4, lower panel). The observed STD NMR signals for GlcNAc moieties also provided important information. In particular, for the LND3 complex, a clear STD NMR intensity was observed for H5 of the terminal GlcNAc at the 6-branch. Alternatively, for LDN6, the H5 signal of the terminal GlcNAc at the 3-branch was also present in the analogous STD NMR spectrum.

The STD NMR analysis of the N-acetyl groups of the GlcNAc and GalNAc moieties was also instrumental to reach additional conclusions on the binding mode. No major signal overlap (Figure 5) took place in this region of the NMR spectrum (δ 1.95–2.0 ppm), and, therefore, the contributions from each acetyl group could be assessed unambiguously. For the nonelongated N-glycan (G0), as discussed above, the acetyl groups of both external GlcNAc moieties showed STD intensity, although larger for that at the 3 arm. For LDN3, only the acetyl group at the 6-branch appeared in the STD NMR spectrum, while for LND6, only the acetyl signal of the 3-branch was observed.

**Figure 6:**
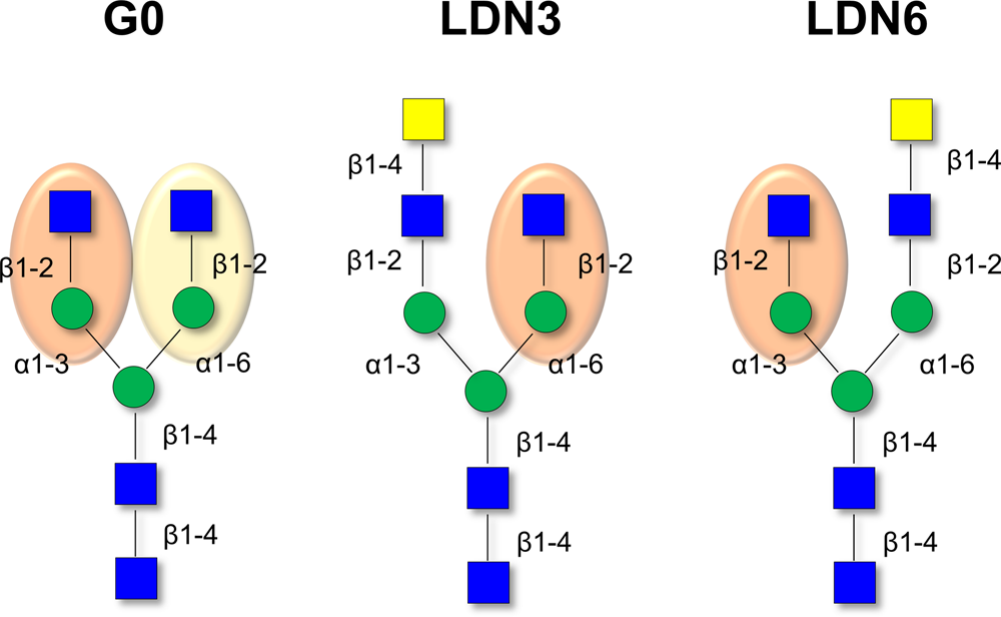
N-glycan binding epitopes recognized by LSECtin. The three N-glycans employed in the study are represented, highlighting the key GlcNAcβ1-Man epitope. For G0, the 1,3-branch (highlighted in pale red) is better recognized than the 1,6-branch (highlighted in pale yellow), according to STD NMR results.

From these data, it is clear that the presence of the terminal GalNAc residue acts as a stop light and precludes binding at that site (Figure 6**)**. The global STD NMR profiles of LDN3 and LDN6 are fairly similar, but the epitope switches from one arm (1–6 for LDN3) to the other (1–3 for LDN6) in solution.

Therefore, the binding features deduced herein for the interaction of glycans G0, LDN3, and LDN6 with LSEctin in solution are strikingly different to those observed for the glycans immobilized on a glycan array.^22^ This fact highlights the dramatic role of glycan presentation for the molecular recognition process. LDN3 is not recognized by LSECtin on the glycan array, but it is indeed recognized in solution with similar affinity as the LDN6 analogue. Recent computational studies^36^ have suggested that galactosylation at the GlcNAc moieties on the two arms of G0 trigger distinct conformational changes. The calculations predicted a 25% increase of the population (from ca. 50% to 75%) of back-folded conformers of the 6-arm over the *N*,*N*′-diacetyl-chitobiose core when galactosylation occurred at the 6 arm, while no such effect was predicted when galactosylation took place at the 3-arm. Nevertheless, although these calculations could provide a hint for a better presentation of the 3-arm in LDN6 to interact with LSECtin, they cannot explain why LDN3 is not recognized in the array setting.

At this point, we can hypothesize that, given its distinct flexibility, the presented GlcNAcβ1-2Man epitope at the 6-arm in LDN3 is hindered when the glycan is attached to a surface, while it remains available for binding in solution. Thus, in order to shed light on the experimental observations, molecular dynamics simulations were carried out to uncover glycan presentation under array conditions.

### Molecular Dynamics Simulations

Under the array conditions, the physical properties of the linker and the solid support determine how the glycan is presented, and thus its availability for receptor binding.^9^ Such heterogeneous conditions are difficult to simulate owing to the lack of information on the exact composition of the support employed, as well as the exact distribution of the glycan conjugating groups (PEGn-NHS on Nexterion Slide H). Even if that information were fully available, the intrinsically amorphous character of polymer coatings precludes the generation of a detailed model of the array conditions. In short, models always include a degree of simplification.

It has been recently demonstrated that the combination of surface and linker polarity are key for determining their degree of contact with the glycan, thus tuning its accessibility.^9^ For this reason, we strived at building a model that correctly reproduces this physical property. The Nexterion Slide H^22^ that was employed in the glycan array experiments consists of a glass slide coated with a hydrophilic polymer, which was here modeled as a cellulose I-beta phase squared slab of 150 by 150 by 20 Å (Figure S13). This approach provides a regular and highly polar surface. The choice of this particular phase was dictated by the regular arrangement of the surface Glc hydroxymethyl groups—roughly perpendicular to the support’s surface—which provides easily accessible anchoring points for the linkers.

Experimentally, the glycans are attached to the solid support through long, polar, and flexible linkers composed of ca. 45 ethylene glycol PEG units (PEG2K-NHS), and a 5-aminopentyl linker at the reducing end that allows irreversible binding through the formation of an amide bond with the NHS group. Using full-sized linkers would create a model computationally unamenable for an all-atom simulation as the ones presented in this work.

Hence, we reduced the linker length preserving its chemical nature (PEG) while exploring a range of linkers’ sizes with lengths up to 40 Å (Figure S14). Further details about the model building and simulation parameters are provided in the Supporting Information.

**Figure 7:**
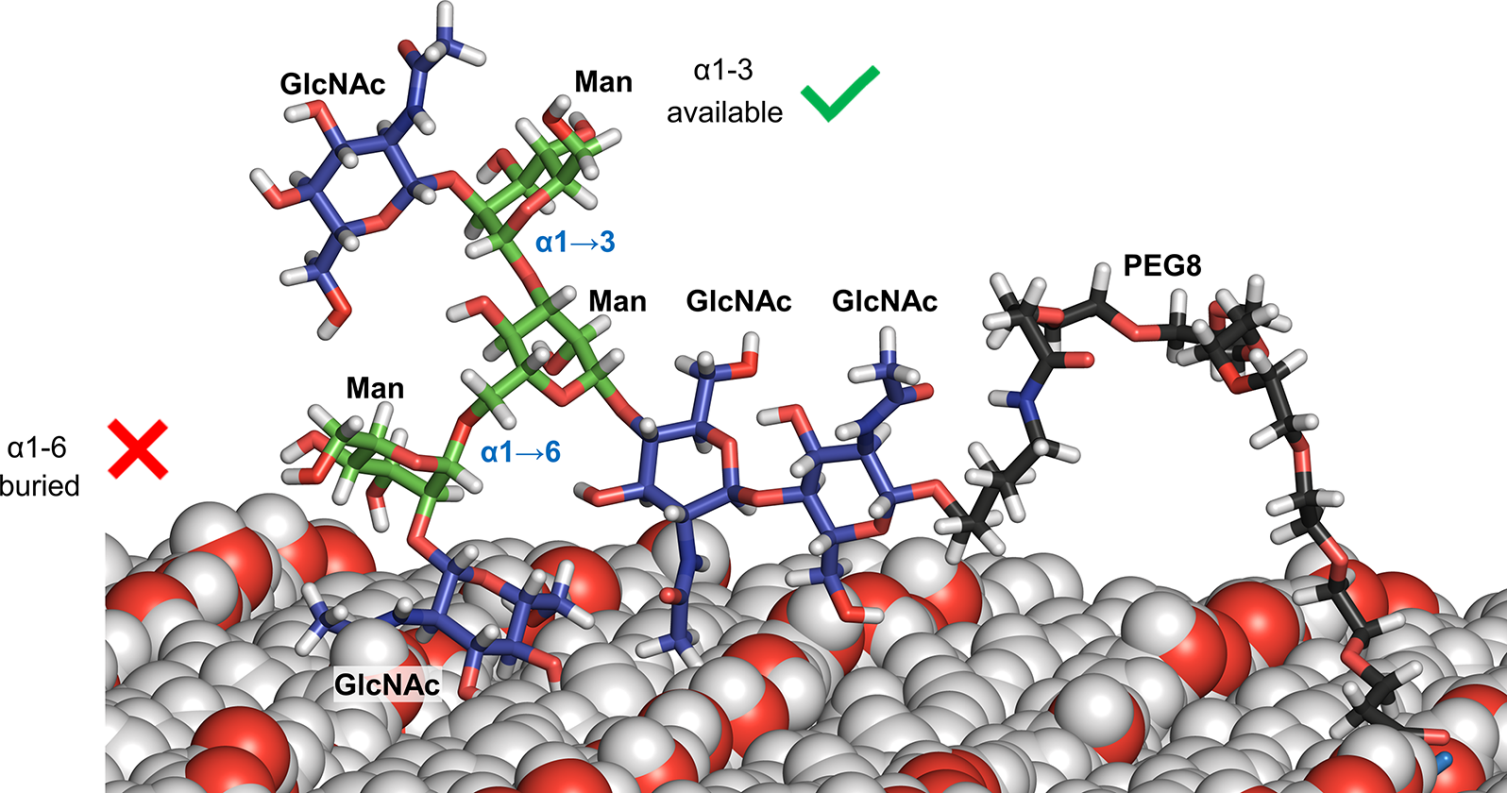
A selected molecular dynamics snapshot showing the 6-arm of the GlcNAcβ1-2Man epitope partially buried at the support’s surface in bCell-PEG8-G0, while the alternative epitope at the 3-arm remains fully accessible.

In this model, a single linker-G0 unit was attached at the center of the slab, and molecular dynamics (MD) simulations were performed to analyze the degree of accessibility of the GlcNAcβ1-2Man epitope at arms 3 and 6. As a representative example, for the simulation involving an 8-unit PEG linker (**bCell-PEG8-G0**), it could be observed that while the 3-arm remains fully exposed to the solvent, and thus available for receptor binding, the 6-arm interacts with the polar surface in such a way that the GlcNAcβ1-Man epitope is partially buried. This fact is expected to translate in a reduced availability for receptor binding (Figure 7).

Indeed, there is an unequal degree of flexibility of the two epitopes: the additional ω angle at the α(1 → 6) linkage provides a superior mobility at the corresponding epitope over that at the α(1 → 3) linkage. Thus, the GlcNAc moiety at the α(1 → 6) arm, which explores a wider conformational space, holds a higher possibility of interacting with the support’s surface, as reflected by its smaller solvent accessible surface area (SASA) compared to that of the α(1 → 3) arm (Figure S15). The established surface-glycan interactions makes the corresponding GlcNAc moiety to be partially buried with respect to that at the α(1 → 3) linkage, which remains fully accessible to receptors.

## Conclusions

Glycan presentation is an essential part of molecular recognition processes. The NMR studies in solution performed herein have confirmed that the GlcNAcβ1-2Man moiety is the minimum epitope recognized by LSECtin. In biantennary N-glycans, GalNAc or Gal substitution at a given arm abolishes recognition at that particular branch. Strikingly, in contrast to the observations made when the same glycans are presented on glycan microarrays, both asymmetric LDN3 and LDN6 N-glycans are similarly recognized by LSECtin in solution. On the glycan array, only LDN6 was recognized by this lectin. The NMR studies unambiguously show that, in the absence of a GalNAc residue (the G0 N-glycan), a certain preference for binding to the 3-arm over the 6-arm is observed in solution (Figure 6). Nevertheless, similar affinities for LDN6 and LDN3 are detected in solution, contrasting with the exquisite specificity observed for binding of the attached glycans on the array.

We hypothesize that, under array conditions, the recognition through the 6-branch is impeded, and the GlcNAcβ1-2Man recognition epitope is accessible to the lectin only when presented on the 3-branch. In solution, both asymmetric glycans are similarly bound, suggesting that in the presence of isotropic motion, the exposed epitopes on both branches are nearly equally accessible. Molecular dynamics simulations on a model surface functionalized with the nonelongated G0 glycan and different linkers support this hypothesis, showing that the 6-arm is more prone to interacting with the surface than the 3-arm, probably due to its intrinsic flexibility, thereby reducing its accessibility for binding to LSEctin.

The results presented herein have general consequences for the molecular recognition field. We^22^ and others^37−39^ have shown through glycan array-based studies that lectin binding toward N-glycans can be highly influenced by the branch position of the recognized main epitope. However, different outcomes can be obtained using diverse experimental approaches. This fact points out the tremendous difficulty of translating *in vitro* results to the *in vivo* environment.^40^ Care should be taken when extracting conclusions from experiments conducted under specific conditions. Molecular recognition details differ from solution state to surfaces. Which one is closer to those existing in nature? Glycans are usually exposed on cell surfaces as part of glycoconjugates forming the glycocalyx. It is tempting to propose that the studies conducted using arrays are closer to those taking place on cell surfaces. However, the architecture of cell glycocalyxes is highly complex,^15,40^ and in this context glycan presentation is difficult to fit in a single, simple, and flat surface presentation model. Also, under immobilized conditions, the presentation of the interacting sugar epitope is essential, and therefore, the length and chemical nature of the linkers used to attach the ligands to surfaces, and the composition of the solid support itself, could also influence the final outcome and the interpretation of the obtained results.^9^

## Supporting Information Available

Materials and methods. Additional NMR experimental data including ligands’ assignment, ^1^H titration, STD-NMR, ROESY-NMR, as well as NOESY-NMR experiments. Additional figures for the bCell-PEGn-G models used in the molecular dynamics simulations (PDF)
Transparent Peer Review report available (PDF)

## Abbreviations

ECD: extracellular domain
CRD: carbohydrate recognition domain.
