# Supplementary material for: Immobilization of Biantennary N-Glycans Leads to Branch Specific Epitope Recognition by LSECtin
Source: ACS Cent Sci. 2022 Sep 20;8(10):1415–23. doi: 10.1021/acscentsci.2c00719 (PMC9615123; doi:10.1021/acscentsci.2c00719)
Supplement: Supplementary file 1 — oc2c00719_si_001.pdf [file oc2c00719_si_001.pdf]

Supplementary Material for:

Immobilization of biantennary N-glycans leads to branch specific epitope recognition by LSECtin

Sara Bertuzzi,[a] Francesca Peccati,[a] Sonia Serna,[b] Raik Artschwager,[b,c] Simona Notova,[d] Michel Thépaut,[d] Gonzalo Jiménez-Osés,[a,e] Franck Fieschi,*[d] Niels C. Reichardt,*[b,f] Jesús Jiménez-Barbero,*[a,e,g,h] and Ana Ardá*[a,e]

[a] CIC bioGUNE, Basque Research & Technology Alliance (BRTA), Chemical Glycobiology group, Bizkaia Technology Park, Building 800, 48160 Derio, Bizkaia, Spain.
[b] CIC biomaGUNE, Glycotechnology Group, Basque Research and Technology Alliance (BRTA), Paseo Miramón 182, 20014 San Sebastian, Spain.
[c] Memorial Sloan Kettering Cancer Center, 417 E 68th St., 10065, New York, NY USA.
[d] University of Grenoble Alpes, CNRS, CEA, Institut de Biologie Structurale, 38000 Grenoble, France.
[e] Ikerbasque, Basque Foundation for Science, Maria Diaz de Haro 3, 48013 Bilbao, Bizkaia, Spain.
[f] CIBER-BBN, Paseo Miramón 182, San Sebastian, Spain.
[g] Department of Organic Chemistry, II Faculty of Science and Technology University of the Basque Country, EHU-UPV, 48940 Leioa, Spain.
[h] Centro de Investigación Biomédica En Red de Enfermedades Respiratorias, Madrid, Spain.

Corresponding Author
* E-mail: jjbarbero@cicbiogune.es
* E-mail: aarda@cicbiogune.es
* E-mail: franck.fieschi@ibs.fr
* E-mail: nreichardt@cicbiomagune.es

KEYWORDS. LSECtin, NMR, lectin, glycan presentation, glycan array

METHODS

LSECtin production, refolding and purification

N-terminal 6-His tagged LSECtin CRD (amino acids 162–292) was cloned downstream to a factor Xa protease site, 3 glycines and with the first cysteine mutated in a serine, into pET-30 plasmid (Novagen) between NdeI and HindIII restriction sites. LSECtin CRD was expressed in E. coli BL21(DE3) at 37°C in 1 L of LB medium supplemented with 50 μg/mL kanamycin. After 3 h of culture (A600nm of 1.7), expression was induced for 3 h at 37°C by the addition of 1 mM isopropyl 1-thio-D-galactopyranoside (IPTG). The protein was expressed in the cytoplasm as inclusion bodies. Cells were harvested by a 20 min centrifugation cycle at 5000 g at 4 °C. The pellet was resuspended in 30 mL of a solution containing 150 mM NaCl, 25 mM Tris-HCl at pH 8, and one antiprotease mixture tablet (Complete EDTA-free, Roche). Cells were disrupted by sonication and cell debris eliminated by ultracentrifugation at 100 000 g for 30 min at 4 °C with a Beckman 45Ti rotor. The pellet was consecutively washed with 2 M urea, 150 mM NaCl, 25 mM Tris-HCl at pH 8 and 1% triton X-100, centrifuged, washed with 150 mM NaCl, 25 mM Tris-HCl at pH 8, centrifuged and solubilized in 30 mL of 6 M guanidine-HCl, 25 mM Tris-HCl at pH 8, 150

mM NaCl, and 0.01% (v/v) β-mercaptoethanol. The mixture was centrifuged at 100 000 g for 45 min at 4 °C, and the supernatant was diluted 5-fold dropwise into 12,5 mM NaCl, 25 mM $CaCl_2$, 25 mM Tris-HCl pH 8 and 10 mM mannose with stirring. The resulting sample was dialyzed against 5 volumes of 25 mM Tris-HCl at pH 8, 150 mM NaCl, and 4 mM $CaCl_2$ (buffer A) with three buffer changes. After dialysis, insoluble precipitate was removed by centrifugation at 100 000 g for 1h at 4 °C.

The supernatant containing the His tagged LSECtin CRD was loaded onto a 1 mL HisTrap HP column (GE Heathcare) at 4 °C. Unbound proteins were washed away with buffer A. LSECtin CRD was eluted with buffer C (150 mM NaCl, 25 mM Tris-HCl at pH 8, 4 mM $CaCl_2$, 0.5 M imidazole). This step was followed by a 125 mL size exclusion chromatography Superose 12 column (GE Heathcare) at 4 °C equilibrated with buffer A. Fractions were analyzed by SDS-PAGE (15%), and LSECtin containing fractions were pooled and concentrated by ultrafiltration (Vivaspin PES, MWCO 5000, from Sartorius).

Ligands synthesis

5-aminopentyl functionalized glycan structures were chemo-enzymatically synthesized as previously described [1,2].

NMR experiments

The NMR experiments were acquired using Bruker AVANCE 2 600 MHz spectrometer (Bruker Inc.; Billerica, MA, US) or Bruker AVANCE 2 800 MHz spectrometer equipped with cryo-probe. All the NMR samples were prepared in 400 μL of total volume and transferred in 5 mm shigemi NMR tubes (New Era Enterprises, Vineland, USA). The pH of the buffer was measured with a Crison Basic 20 pH meter (Crison Instruments SA, Barcelona, Spain) and adjusted with the required amount of NaOH and HCl or NaOD and DCl.

^{1}H-NMR titration

The NMR experiments for the disaccharide and the non-elongated N-glycan were acquired using 600 MHz spectrometer. LSECtin CRD was employed at a concentration of 120 μM in deuterated buffer composed as follow: tris-d11 25 mM, NaCl 150 mM, $CaCl_2$ 4 mM, dithiothreitol-d10 (DTT-d10) 2 mM at pD 8.4. At each protein:ligand ratio during the titration (1:0, 1:1, 1:2, 1:5, 1:10 and 1:20) an ^{1}H experiment was acquired. The concentration of the ligand at the last point (ratio 1:20) was 2.4 mM. The sequence zgesgp was selected from Bruker's library. The acquisition was done at 298K, and the experiment was set with 32 number of scans.

The ^{1}H titration experiments for LDN3 and LDN6 were acquired using 800 MHz spectrometer. LSECtin CRD was employed at a concentration of 82 μM in deuterated buffer composed as follow: tris-d11 25 mM, NaCl 150 mM, $CaCl_2$ 4 mM, dithiothreitol-d10 (DTT-d10) 2mM at pD 8.4. At each protein:ligand ratio during the titration (1:0, 1:0.5, 1:1, 1:2, 1:4, 1:6 and 1:34) an ^{1}H experiment was acquired with 64 number of scans. The concentration of the ligand at the last point (ratio 1:34) was 2.78 mM. The sequence zgesgp was selected from Bruker's library and the acquisition was done at 298K.

STD NMR experiments

The STD sequence sddiffesgp.3 was employed from Bruker's library for all the experiments and the temperature during the acquisition was 310K. The on-resonance frequency was set for at δ 0.83 and 0.6 for the aromatic irradiation and at δ 6.8 for the aliphatic irradiation. The off-resonance frequency was set at 100 ppm. The on- and off-resonance spectra were registered in an interleaved mode with the same number of scans. The STD NMR spectra were obtained by subtracting the on-resonance spectrum from the off-resonance spectrum and the STDD NMR spectra were obtained by subtracting to the STD spectrum of the protein/ligand mixture the STD spectrum obtained with the protein alone. The STD Amplification Factor (STD-AF) and the percentage of STD (STD%) were calculated on the basis of the STD spectra. Reference experiments were acquired on samples containing only the protein as well as only the ligands under the same experimental conditions to verify the authenticity of the binding.

The STD sample for LSECtin and Disaccharide was prepared with LSECtin CRD 120 μM in deuterated buffer (Tris-d11 25 mM, NaCl 150 mM, $CaCl_2$ 4 mM, dithiothreitol-d10 (DTT-d10) 2mM at pD 8.4) and the disaccharide concentrated 8.4 mM (ratio lectin:ligand = 1:70). The experiment was acquired with 600 MHz spectrometer and the parameters were optimized with 1024 number of scans, 2 seconds of saturation time and 2 seconds of relaxation delay. The STD sample for LSECtin and the N-glycans was prepared with LSECtin CRD 120 μM in deuterated buffer (Tris-d11 25 mM, NaCl 150 mM, $CaCl_2$ 4 mM, dithiothreitol-d10 (DTT-d10) 2mM at pD 8.4) and the respective N-glycan at each 4.08 mM (ratio lectin:ligand = 1:34). The experiment was acquired with 800 MHz spectrometer and the parameters were optimized with 2880 number of scans, 2 seconds of saturation time and 15 seconds of relaxation delay. The spin-lock filter used to remove the NMR signals of the macromolecule was set at 40 ms.

Molecular dynamics simulations

The cellulose I-beta slab used as hydrophilic solid support (Figure S13) was generated using the Cellulose-Builder web server.[3] With this slab, five models of supported glycans were built with varying length of the PEG linker to explore the effect of different glycan/support separations on the availability of the α(1→3) and α(1→6) branches. These models are named according to the number of units of the PEG linker connecting the glycan to the solid support: bCell-PEG0-G0, bCell-PEG2-G0, bCell-PEG6-G0, bCell-PEG8-G0 and bCell-PEG10-G0, corresponding to 0, 2, 6, 8 and 10 ethylene glycol units, respectively (Figure S14).

Molecular dynamics simulations were carried out with the AMBER 20 suite [4] using the GLYCAM 06j-1 [5] force field for G0 and the cellulose support and GAFF2 [6] for the linkers. The models were then immersed in a water box of TIP3P water molecules [7] with an 8 Å buffer distance from the solute. No neutralizing counterion was needed as all the systems are neutral. A two-stage geometry optimization approach was performed. The first stage minimizes only the positions of solvent molecules, and the second stage is an unrestrained minimization of all the atoms in the simulation cell. The systems were then heated by incrementing the temperature from 0 to 300 K under a constant pressure of 1 atm and periodic boundary conditions. Harmonic restraints of 10 kcal mol^{-1} $Å^{-2}$ were applied to the solute, and the Andersen temperature coupling scheme [8] was used to control and equalize the temperature. The time step was kept at 1 fs during the heating stages, allowing potential inhomogeneities to self-adjust.

The SHAKE algorithm was employed for further equilibration and production with a 2 fs time step. [9] Long-range electrostatic effects were modeled using the particle mesh Ewald method. [10] A cutoff of 8 Å was applied to Lennard-Jones interactions. Each system was equilibrated for 2 ns at constant volume and temperature of 300 K. Production simulations were run as a 200 ns trajectory for each model within the NVT ensemble. To guarantee the structure integrity of the cellulose slab while allowing for some flexibility to mimic the unstructured nature of the hydrophilic polymer coating, 10 kcal mol^{-1} $Å^{-2}$ harmonic restraints were applied to the first and last residue of each cellulose strand along the whole simulations. For system bCell-PEG8-G0 28 independent trajectories of 60 ns each were run in order to enhance conformational sampling, amounting to an accumulated simulation time of 1680 ns. The solvent-accessible surface areas (SASA) of the terminal GlcNAc units were measured from the aggregated trajectory using the surf command of the cpptraj tool in AMBER. SASA values were computed every 20 ps, collecting 84,000 data points in total for each GlcNAc unit.

Kinetic parameters estimation

2D NOESY Experiments

2D-NOESY experiments were acquired using Bruker AVANCE 2 800 MHz spectrometer equipped with cryoprobe. All experiments were acquired at 298 K with an in-house NOESY sequence using a mixing time of 0 and 100 msec and 32 scans. The samples were prepared in deuterated phosphate-buffered saline (buffer (Tris-d11 25 mM, NaCl 150 mM, $CaCl_2$ 4 mM, dithiothreitol-d10 (DTT-d10) 2mM at pD 8.4). The protein:ligand ratio was set at 1:10, being LSECtin at a concentration of 120 µM.

EXSY Calculation

The analysis of the exchange peaks, which display opposite sign to the ROE-derived peaks, allowed the calculation of the kinetic exchange rates k_{on} and k_{off} (Figure S16). Once the exchange cross peaks were identified, the corresponding kinetic data were estimated with the EXSY analysis for the complexes LSECtin:disaccharide and LSECtin:G0 [11,12]. Two 2D-NOESY (0 and 100 ms) were recorded for each sample and after integration of the cross- and diagonal-peaks, the measured intensities were analysed using the ExsyCalc software to deduce the pseudo-first-order exchange rates (k_{on} and k_{off}), reported in Figure S17. Both rates are faster for the disaccharide, a fact that leads to similar affinities, although the binding kinetics are different.

References:

[1] Echeverria, B.; Serna, S.; Achilli, S.; Vivès, C.; Pham, J.; Thépaut, M.; Hokke, C.H.; Fieschi, F.; Reichardt, N.-C. Chemoenzymatic Synthesis of N-glycan Positional Isomers and Evidence for Branch Selective Binding by Monoclonal Antibodies and Human C-type Lectin Receptors. *ACS Chem. Biol.* **2018**, *13*, 2269–2279, doi:10.1021/ACSCHEMBIO.8B00431.

[2] Serna, S.; Etxebarria, J.; Ruiz, N.; Martin-Lomas, M.; Reichardt, N.C. Construction of N-Glycan Microarrays by Using Modular Synthesis and On-Chip Nanoscale Enzymatic Glycosylation. *Chem. – A Eur. J.* **2010**, *16*, 13163–13175, doi:10.1002/CHEM.201001295.

[3] Gomes, T. C. F.; Skaf, M. S. Cellulose-Builder: A Toolkit for Building Crystalline Structures of Cellulose. *J. Comput. Chem.* **2012**, *33*, 1338–1346, doi:10.1002/jcc.22959.

[4] Götz, A.W.; Williamson, M.J.; Xu, D.; Poole, D.; Le Grand, S.; Walker, R.C. Routine Microsecond Molecular Dynamics Simulations with AMBER on GPUs. 1. Generalized Born. *J. Chem. Theory Comput.* **2012**, *8*, 1542–1555, doi: 10.1021/ct200909j.

[5] Kirschner, K.N.; Yongye, A.B.; Tschampel, S.M.; González-Outeiriño, J.; Daniels, C.R.; Foley, B.L; J. Woods, R. GLYCAM06: A Generalizable Biomolecular Force Field. Carbohydrates *J. Comput. Chem.* **2007**, *29*, 622-655, doi: 10.1002/jcc.20820.

[6] Wang, J.; Wolf, R.M.; Caldwell, J.W.; Kollman, P.A.; Case, D.A. Development and Testing of a General Amber Force Field. *J. Comput. Chem.* **2004**, *25*, 1157-1174, doi: 10.1002/jcc.20035.

[7] Jorgensen, W.L.; Chandrasekhar, J; Madura J.D.; Comparison of Simple Potential Functions for Simulating Liquid Water,. *J. Chem. Phys.* **1983**, *79*, 926, doi:10.1063/1.445869.

[8] Andersen, H.C. Molecular Dynamics Simulations at Constant Pressure and/or Temperature. *J. Chem. Phys.* **1980**, *72*, 2384, doi: 10.1063/1.439486.

[9] Miyamoto , S.; Kollman, P.A. Settle: An Analytical Version of the SHAKE and RATTLE Algorithm for Rigid Water Models. *J. Comput. Chem.* **1992**, *13*, 952-962, doi:10.1002/jcc.540130805.

[10] Darden, T.; York, D.; Pedersen Particle mesh Ewald: An N·log(N) Method for Ewald Sums in Large Systems. *J. Chem. Phys.* **1993**, *98*, 10089, doi:10.1063/1.464397.

[11] Perrin, C.L.; Dwyer, T.J. Application of two-dimensional NMR to kinetics of chemical exchange. *Chem. Rev.* **2002**, *90*, 935–967, doi:10.1021/CR00104A002.

[12] Latham, M.; Zimmermann, G.; Pardi, A. NMR chemical exchange as a probe for ligand-binding kinetics in a theophylline-binding RNA aptamer. *J. Am. Chem. Soc.* **2009**, *131*, 5052–5053, doi:10.1021/JA900695M.

SUPPLEMENTARY FIGURES

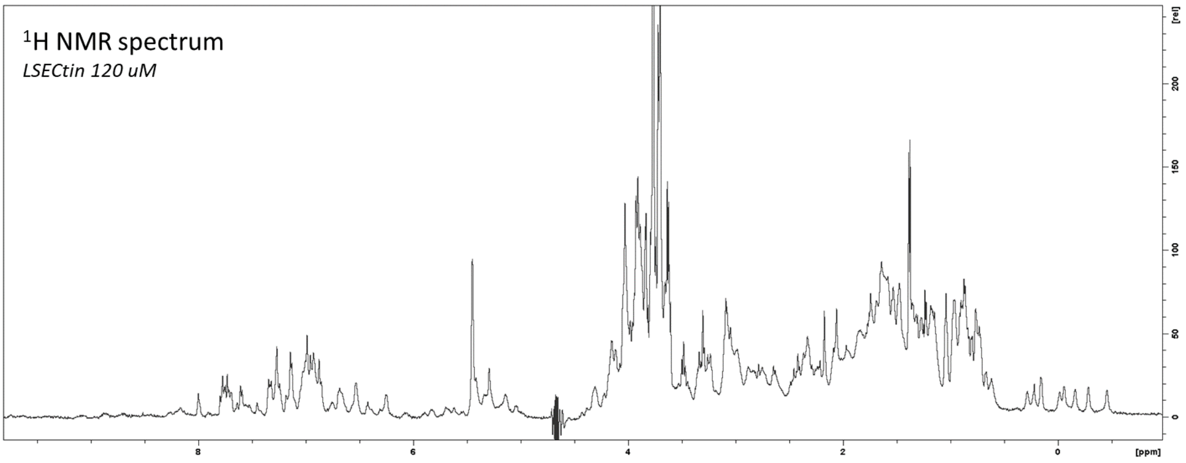

Figure S1. *¹H NMR spectrum of LSECtin.* Spectrum acquired at 298 K with 32 scans. Buffer employed: TRIS 25 mM, NaCl 150 mM, $CaCl_2$ 4 mM, DTT 2 mM, pH 8.4. From the ¹H NMR analysis of the protein LSECtin in solution without ligands, it is possible to appreciate a good chemical shift dispersion, meaning that the tertiary structure of the protein is intact. Moreover, the protein is stable between 288 and 310 K (data not shown).

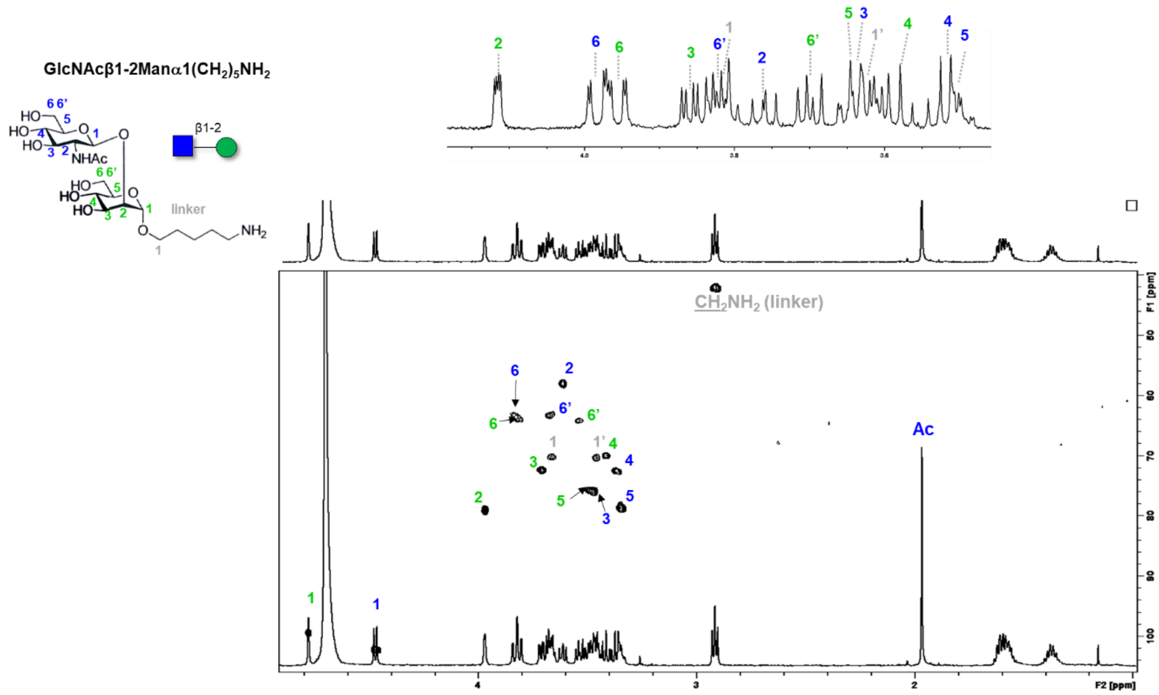

Figure S2. *¹H and ¹H-¹⁵N HSQC assignment of the disaccharide GlcNAcβ1-2Man.* The color code used for the assignment is based on the colors of the monosaccharides in the SNFG representation reported on the top left.

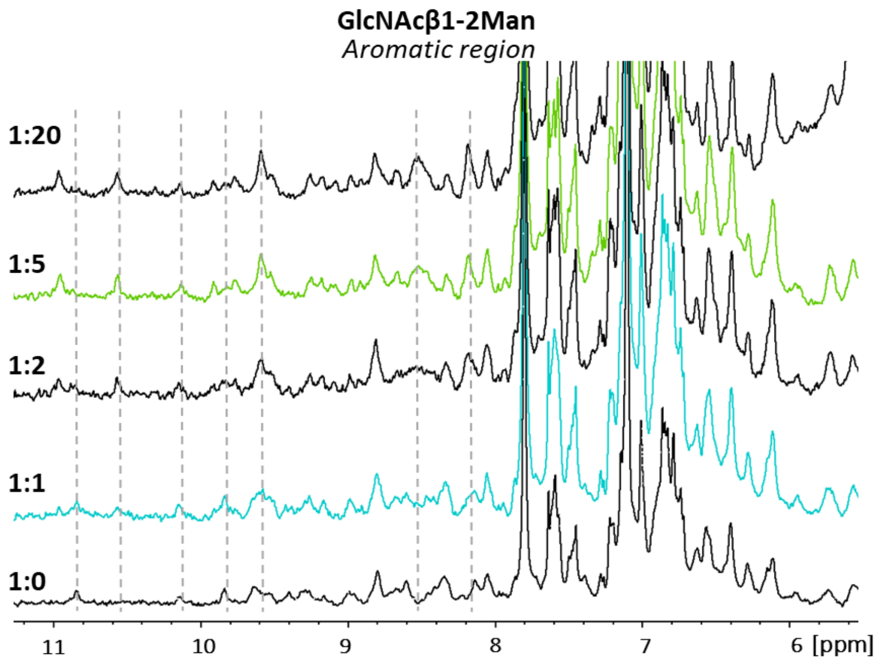

Figure S3 *LSECtin:disaccharide titration*. Stacked ¹H-NMR spectra acquired during the titration of disaccharide GlcNAcβ1-2Man to a sample containing LSECtin CRD (120 μM in deuterated buffer). The relative lectin:ligand ratios are reported above each spectrum. Expansion of the low field region of the spectra (aromatic region) with signal suffering strong changes during the titration highlighted.

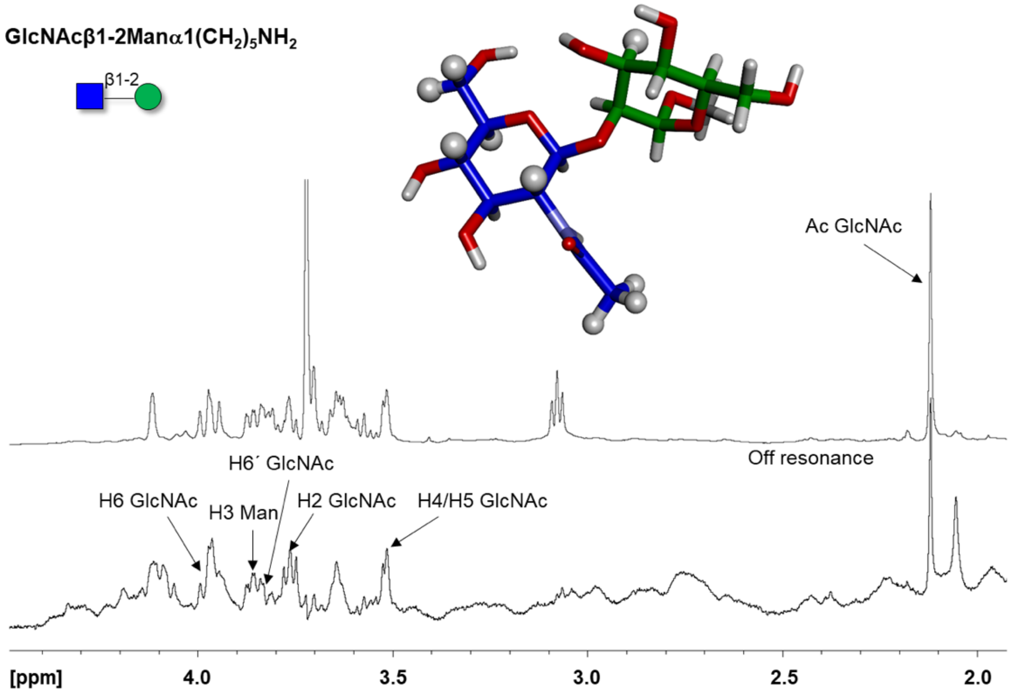

Figure S4. *STD LSECtin and the disaccharide GlcNAcβ1-2Man*. Off-resonance spectrum and STD spectrum obtained with aliphatic irradiation. Annotations of the main ^{1}H signals are reported. The irradiation of the STD spectrum was set at δ 0.83 ppm and lectin:ligand molar ratio employed was 1:70 (being LSECtin CRD at a concentration of 120 μM). The STD experiments were acquired with 2 seconds of saturation time and 5 seconds of relaxation delay at 310K. On top figure: 3D representation of the disaccharide.

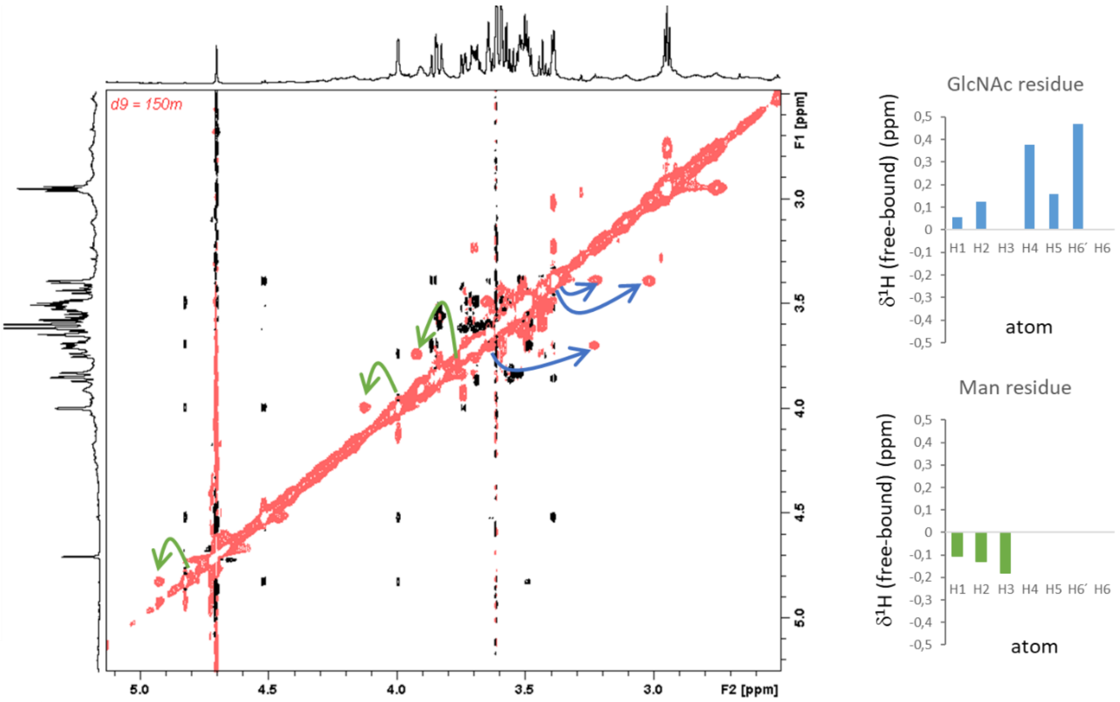

Figure S5. *2D ROESY NMR of LSECtin and the disaccharide GlcNAcβ1-2Man*. Lectin:ligand molar ratio employed was 1:10 (being LSECtin CRD at a concentration of 120 μM). ROESY-NMR experiment acquired with 150 ms of mixing time. Chemical exchange-mediated crosspeaks of the ligand protons are pointed with arrows. On the right, δ^{1}H ppm difference (free-bound) for each proton of the two sugar moieties.

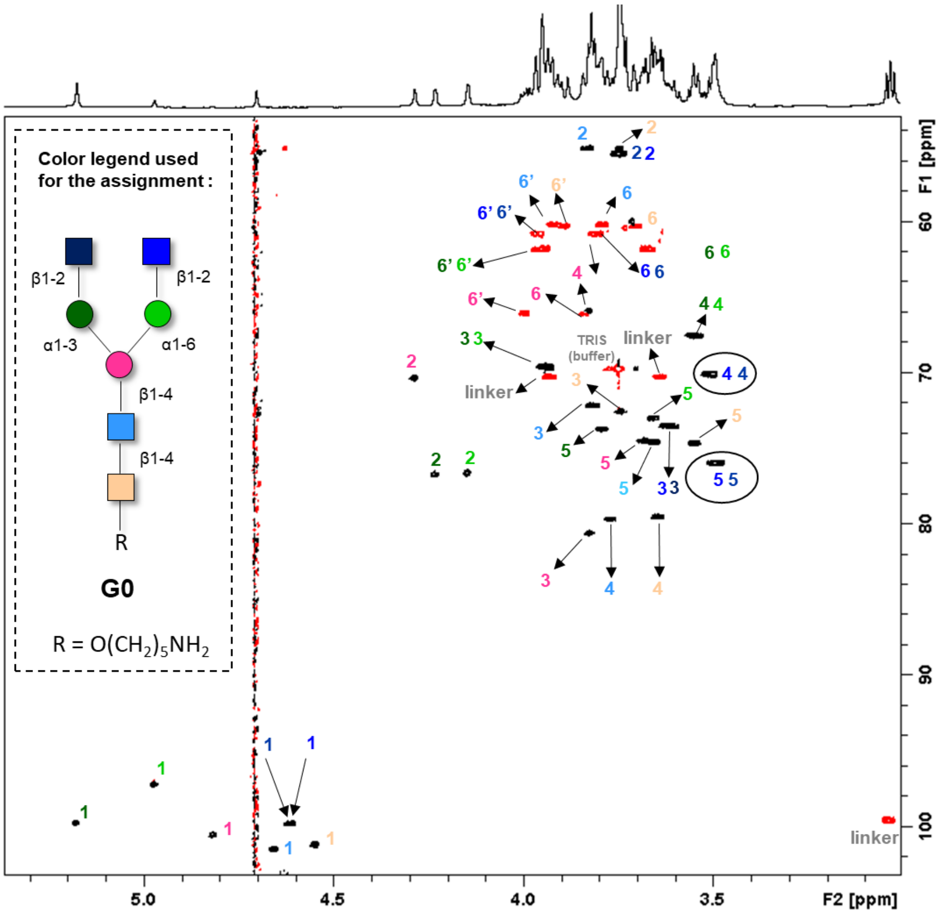

Figure S6. *¹H-¹⁵N HSQC assignment of N-glycan G0.* The color code used for the assignment is based on the legend reported on the top left of the figure.

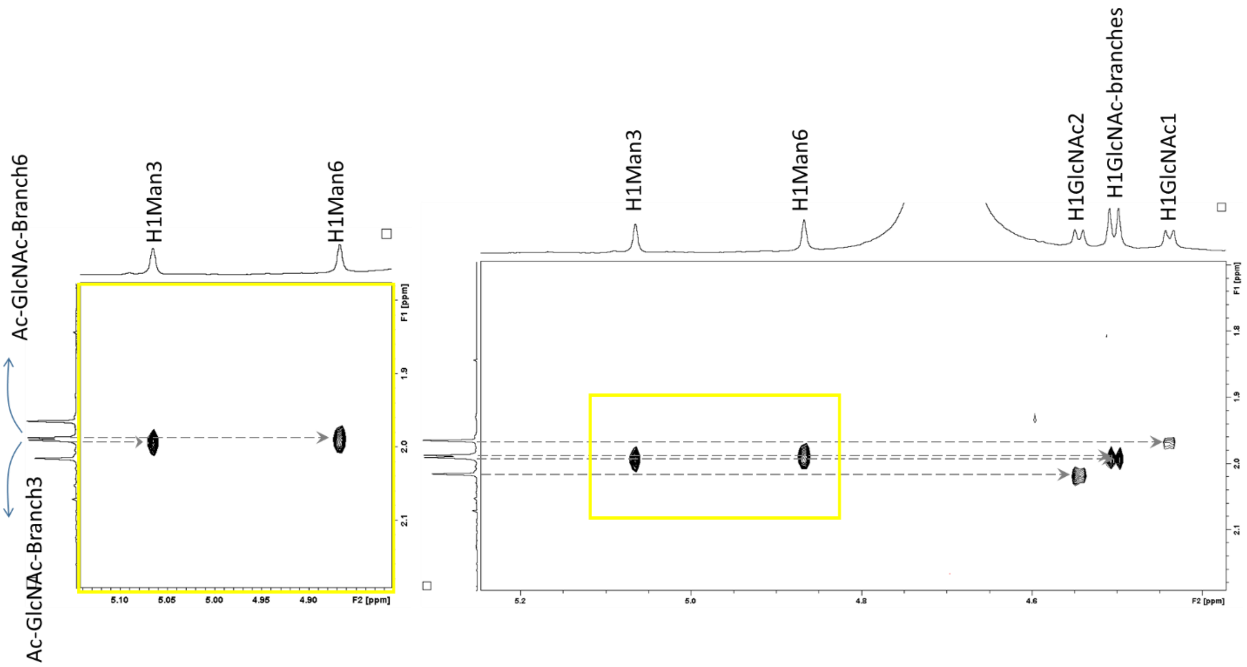

Figure S7. *Justification of Acetyl groups assignment for G0 through NOE experiment.* NOE-NMR correlation between the proton resonances of the acetyl groups of the two terminal GlcNAc residues and the anomeric proton of the Man residue at either the 3- and 6-branch of G0 N-glycan.

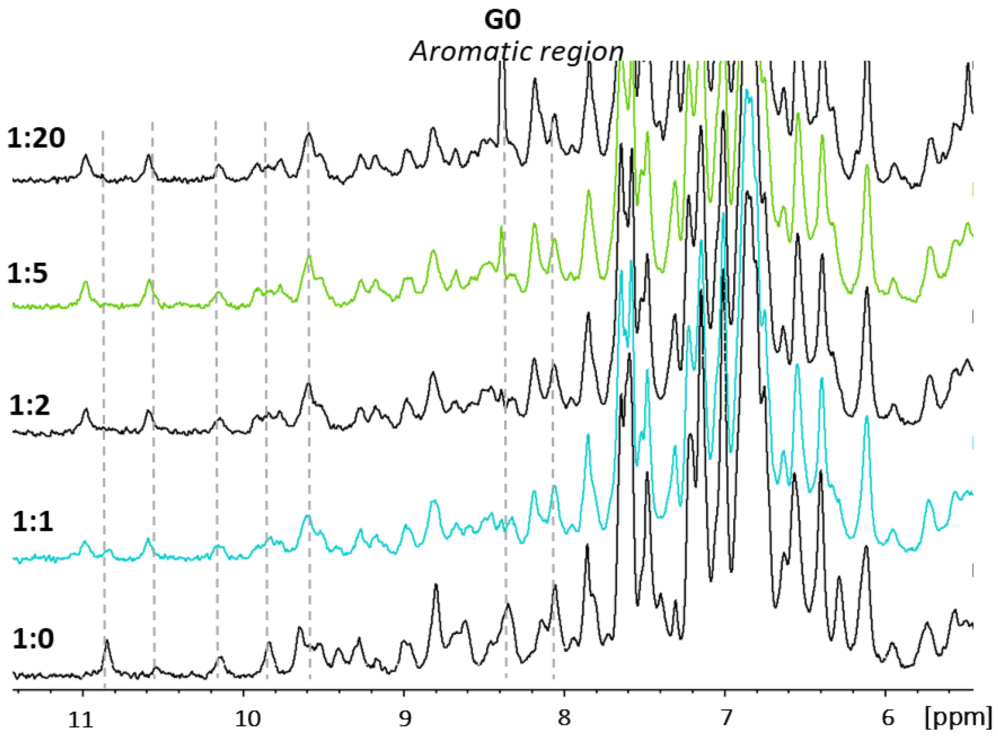

Figure S8. *LSECtin:non-elongated N-glycan (G0) titration*. Stacked ^{1}H-NMR spectra acquired during the titration of G0 to a sample containing LSECtin CRD (120 μM in deuterated buffer). The relative lectin:ligand ratios are reported above each spectrum. Expansion of the low field region of the spectra (aromatic region) with signal suffering strong changes during the titration highlighted.

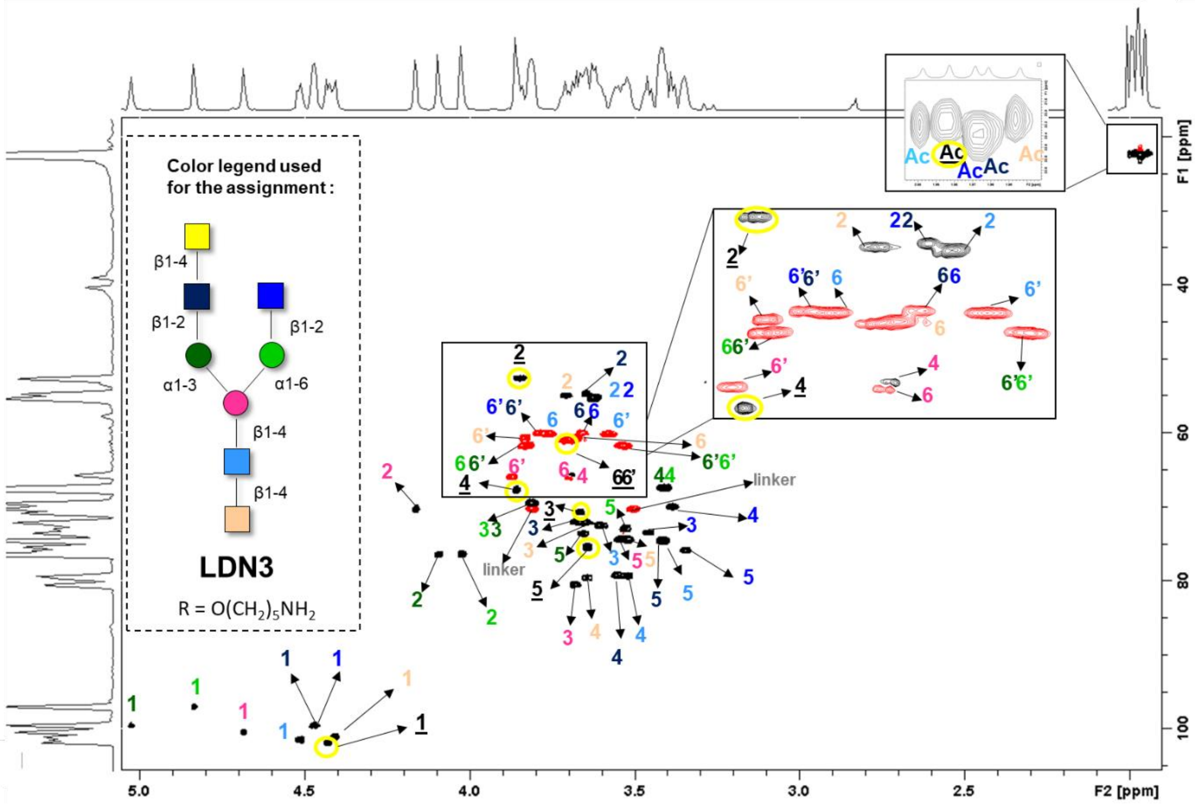

Figure S9. *¹H-¹⁵N HSQC assignment of N-glycan LDN3*. The color code used for the assignment is based on the legend reported on the top left of the figure

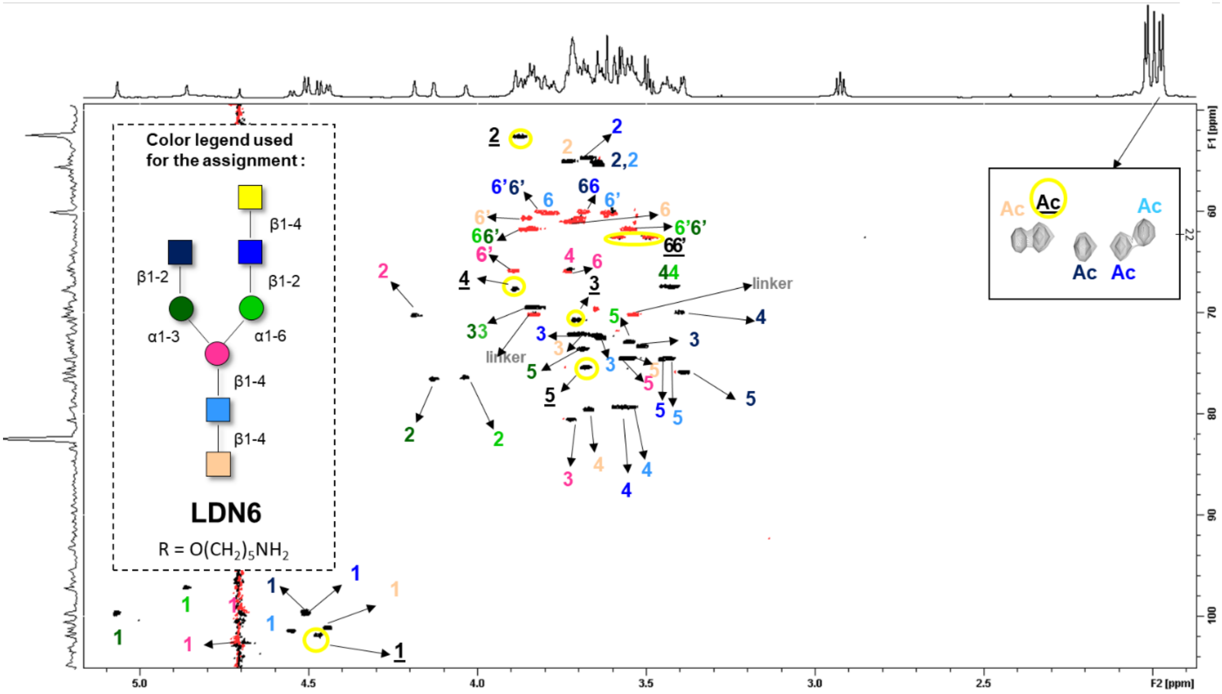

Figure S10. ¹H-¹⁵N HSQC assignment of N-glycan LDN6. The color code used for the assignment is based on the legend reported on the top left of the figure

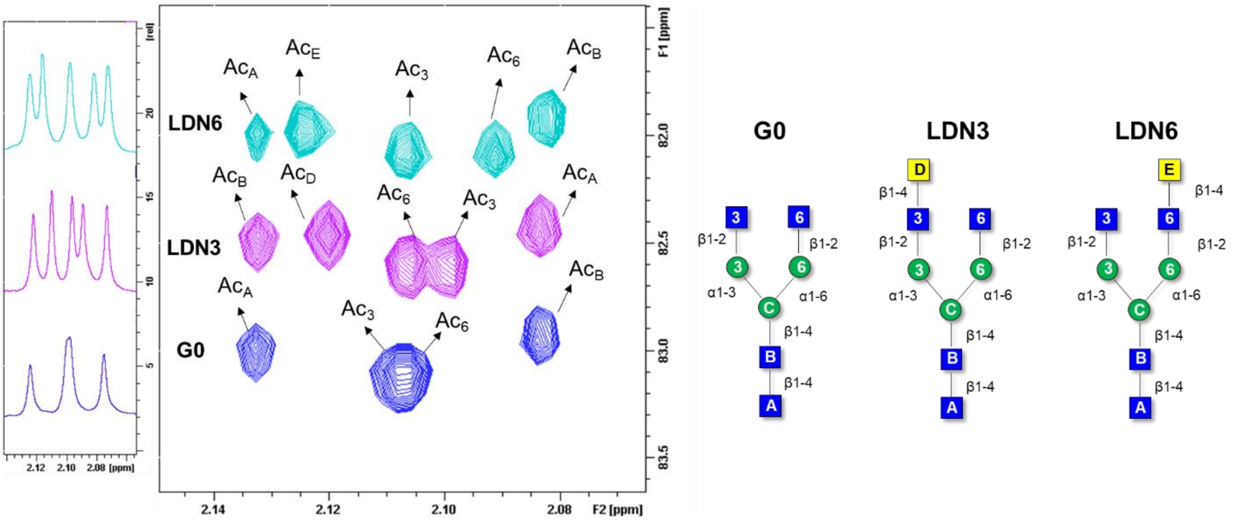

Figure S11. *Close-up to the ¹H-¹⁵N HSQC assignment of N-acetyls groups of G0, LDN3 and LDN6.*

Nomenclature used for the acetyl groups assignment reported on the right.

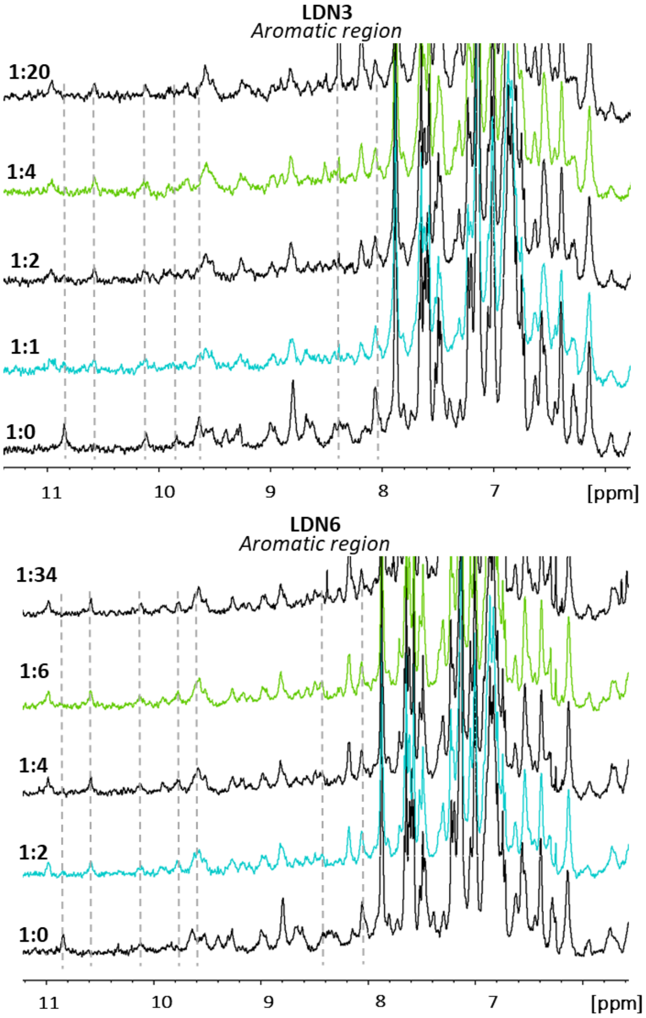

Figure S12. *LSECtin:LDN3/LDN6 N-glycan titration*. Stacked ^{1}H-NMR spectra acquired during the titration of asymmetric N-glycans (on the top LDN3 and on the bottom LDN6; amounts of equivalents reported for each spectrum). LSECtin CRD was employed at a concentration of 82 μM in deuterated buffer. Expansion of the low field region (aromatic region) of the spectra with signal suffering strong changes during the titration highlighted.

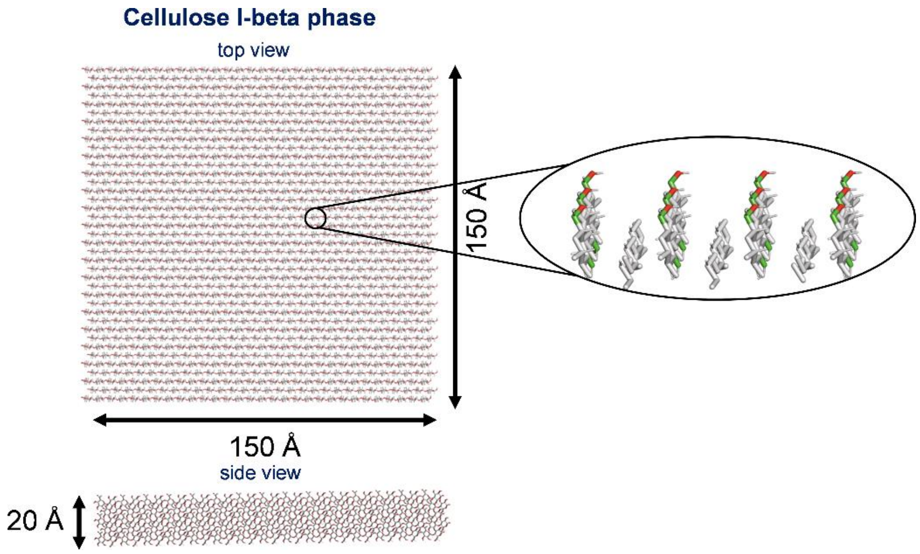

Figure S13. Top and side views of the cellulose slab used as model for the solid support, with indication of the hydroxymethyl groups (in green) used to tether the linkers to the surface.

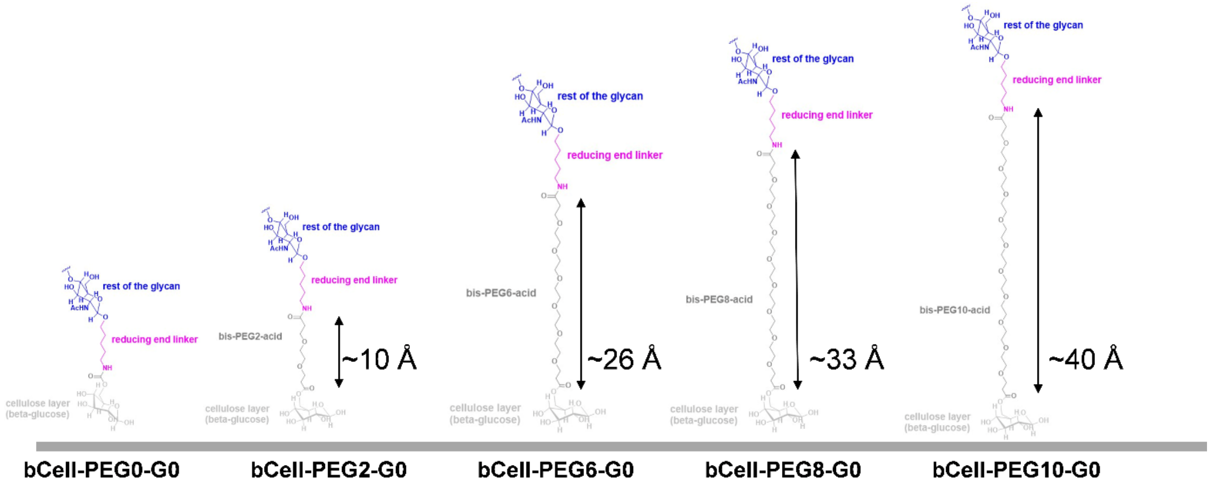

Figure S14. Composition of the five bCell-PEGn-G models (n=0, 2, 6, 8, 10) used in the simulations, with indication of the lengths of the fully extended linkers.

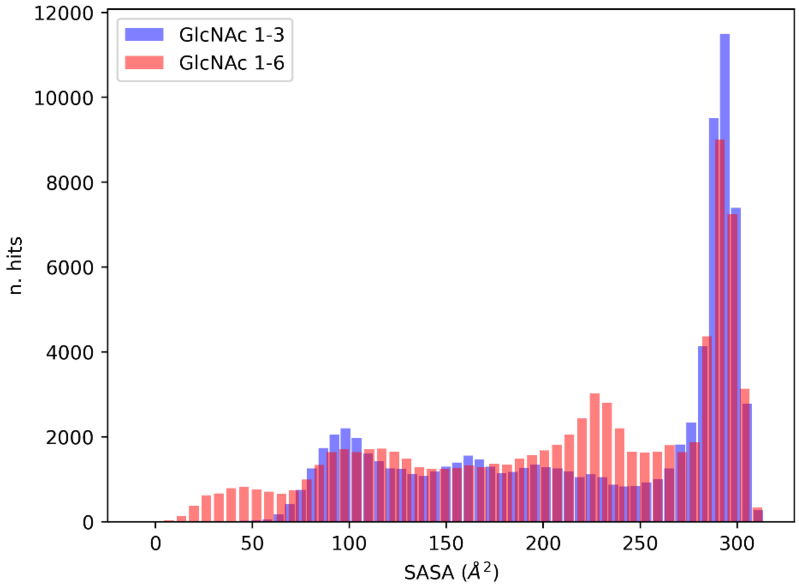

Figure S15. Distribution of SASA values for the terminal GlcNAc units at the α(1→3) (blue) and α(1→6) (red) arms of the **bCell-PEG8-G0** model accumulated over 28 independent 60 ns MD simulations. SASA values are distributed asymmetrically among the two carbohydrates, with the less accessible α(1→6) one showing higher population at low (0-50 Å) and medium (175-260) SASA values (*i.e.* buried) and the more accessible α(1→3) one showing higher population at high SASA values (275-300 Å) (*i.e.* exposed).

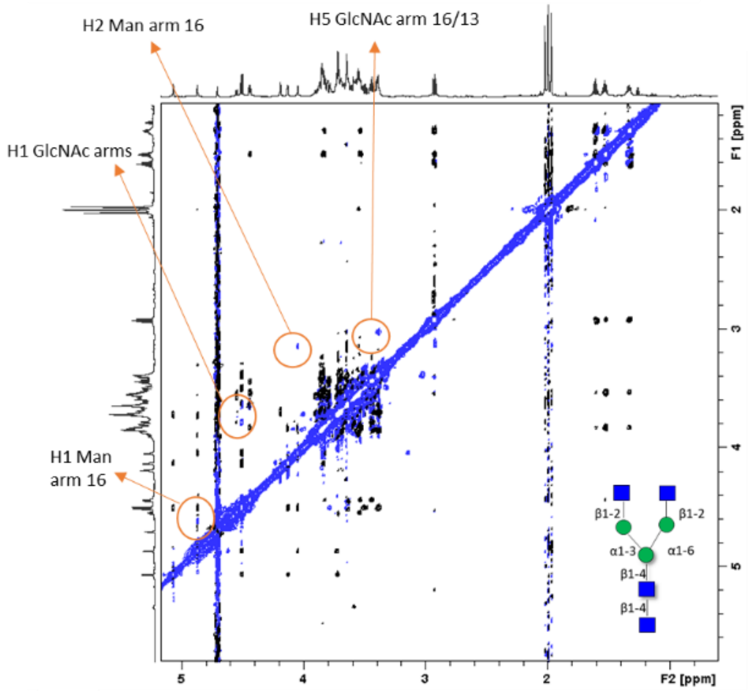

Figure S16. *2D ROESY NMR of LSECtin and G0 glycan*. Lectin:ligand molar ratio employed was 1:10 (being LSECtin CRD at a concentration of 120 µM). ROESY-NMR experiment acquired with 150 ms of mixing time. Chemical exchange-mediated crosspeaks of the ligand protons are pointed with arrows.

LSECtin and G0

EXSY calc	k_{off} (s^{-1})
H1 Man (arm 1-6)	0.211
H2 Man (arm 1-6)	0.140
H5 GlcNAc (16 or 13)	0.276
**AVERAGE k_{off}**	**0.209**

Extimated K_D (*Powlesland at al, 2007 – competition assay*) = 2.6 (µM)
k_{off} = 0.209 (s^{-1})
K_{on}= 80384 ($M^{-1} * s^{-1}$)

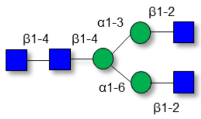

K_D from literature
k_{off} Determined from EXSY
k_{on} Calculated from K_D and k_{off} ($K_D = k_{off}/k_{on}$)

LSECtin and GlcNAcβ1-2Man

EXSY calc	k_{off} (s^{-1})
H1 GlcNAc	1.091
H1 Man (arm 1-6)	1.041
H2 Man (arm 1-6)	0.654
**AVERAGE k_{off}**	**0.928**

Extimated K_D (*Powlesland at al, 2007 – competition assay*) = 3.5 (µM)
k_{off} = 0.928 (s^{-1})
K_{on}= 265142 ($M^{-1} * s^{-1}$)

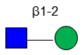

Kon and Koff values are faster for the disaccharide

Figure S17. *EXSY Calculation for LSECtin/Disaccharide and LSECtin/G0 systems.* Both rates are faster for the disaccharide.
