# Supplementary material for: Immobilization of Biantennary N-Glycans Leads to Branch Specific Epitope Recognition by LSECtin
Source: ACS Cent Sci. 2022 Sep 20;8(10):1415–23. doi: 10.1021/acscentsci.2c00719 (PMC9615123; doi:10.1021/acscentsci.2c00719)
Supplement: Supplementary file 2 — oc2c00719_si_002.pdf [file oc2c00719_si_002.pdf]

oc-2022-00719g.R1

Name: Peer Review Information for "Immobilization of biantennary N-glycans leads to branch specific epitope recognition by LSECtin"

First Round of Reviewer Comments

Reviewer: 1

Comments to the Author

This is an important piece of work as the study experimentally shows that immobilization of glycan chains on surfaces, e.g., glycan micro arrays can have a substantial influence on the mode of presentation of glycans. It is shown that, arguably by interacting with the array/chip surface, the 1,6-arm of a biantennary N-glycan cannot be recognized by a cognate lectin, LSEctin. Using simple ^{1}H NMR spectra and STD NMR experiments the authors show that in solution, where the 1,6-arm is not buried on an artificial surface, recognition of the β-GlcNAc-(1,2)-α-D-Man moiety is not obstructed. The study demonstrates that uncritical interpretation of results from glycan micro arrays may be misleading and suggests that results from glycan array binding experiments need less biased and more detailed follow-up assays. NMR spectroscopy lends itself well for such a purpose and can be considered a goldstandard when it comes to judging between "binding" and "no binding". The experiments have been done with care and the results are clearly presented. The manuscript can be essentially published as it is except for some minor points that I list in the following.

Figure 1: At least a link to the nomenclature/symbols used for the monosaccharide units should be given. A key with the Figure would be helpful for non-glycobiologist.

Figures 2 and S3: For the non-specialist in protein-NMR it would be nice to have an assignment of the different signal groups as aliphatic, aromatic etc. in Fig. S3. Looking at Figure 2 very critically, one identifies an additional signal in the LDN6 bound species at a ratio of 1:34. Of all spectra shown, it is only seen in this one spectrum. Any hint where this comes from? Wouldn't it make sense to also quote Figs. S8 and S12 in the text referring to Fig. 2?

p. 4 and Figure S5: This is an interesting finding. Exchange is slow on the chemical shift time scale and at the same time saturation transfer is observed due to slow exchange on the relaxation time scale of the free ligand. Does this information maybe enable the authors to estimate an upper limit for the off-rate constant?

Figure 3: Please mention the saturation time and the number of scans (experiment time) in the legend. The nomenclature is not clear. In the spectra Man residues are labeled "C", "D", "E" whereas the cartoon to the right has "C", "3", "6". Maybe I have been missing the key to this, but I couldn't find it.

p. 6: "Interestingly, the STD-NMR signals for the acetyl groups were always remarkably stronger under aliphatic irradiation, suggesting that the methyl groups are close to aliphatic residues of the protein." Can direct irradiation be excluded?

Figure 6: Why are the two arms of different color?

MD simulations: It would be nice to have some details. Or did I misunderstand it and the discussion refers to published data only?

Reviewer: 2

Comments to the Author

Glycan array screening as become the dominant method for determining glycan-protein specificity. However, as this paper clearly demonstrates, immobilizing the glycan can alter the ability of the glycan to bind to a given protein. This is a very often over-looked issue, and this paper provides a compelling example of this phenomenon.

There are a few issues that should however be addressed that would push the technical quality into the top 1%.

1) The authors should confirm that the printed glycan in the array is not mis-printed or otherwise incorrect. This may be confirmed by demonstrating that the LDN3 ligand on the array binds as expected to other proteins. Array printing errors and other issues could otherwise explain the observation.

2) The authors illustrate with a figure the accessibility of the 1-3 arm of the glycan in the MD simulation snapshot. However, they need to quantify this accessibility over the course of the simulation, and similarly quantify the level of accessibility for the 1-6 arm.

3). I urge the authors not to refer to G0 as a symmetric glycan. Because the core contain 1-3 and 1-6 linkages, it is NOT symmetric and this word choice rather goes against their efforts to present the idea that these arms are not actually equivalent. This is an important point because the use of the SNFG symbols tends to further imply symmetry.

Author's Response to Peer Review Comments:

Dear Prof. Editor,

Many thanks for your letter regarding our manuscript oc-2022-00719g, submitted for publication in ACS Central Science,

We are indeed very pleased for the comments of the reviewers. Moreover, these comments have helped us to provide a new version of the manuscript, considering all the points raised by the them. Many thanks to them for their constructive attitude.

We enclose the detailed point-by-point answers as a separate file.

We also enclose the new version of the manuscript, both with highlights in yellow showing the modifications and without highlights.

We hope that this new version might now be acceptable for publication,

Looking forward to hearing from you, I remain

With my warmest regards,

Jesús

Reviewer 1

This is an important piece of work as the study experimentally shows that immobilization of glycan chains on surfaces, e.g., glycan micro arrays can have a substantial influence on the mode of presentation of glycans. It is shown that, arguably by interacting with the array/chip surface, the 1,6-arm of a biantennary N-glycan cannot be recognized by a cognate lectin, LSEctin. Using simple ^{1}H NMR spectra and STD NMR experiments the authors show that in solution, where the 1,6-arm is not buried on an artificial surface, recognition of the b- GlcNAc-(1,2)-a-D-Man moiety is not obstructed. The study demonstrates that uncritical interpretation of results from glycan micro arrays may be misleading and suggests that results from glycan array binding experiments need less biased and more detailed follow-up assays. NMR spectroscopy lends itself well for such a purpose and can be considered a gold- standard when it comes to judging between "binding" and "no binding". The experiments have been done with care and the results are clearly presented. The manuscript can be essentially published as it is except for some minor points that I list in the following.

1) Figure 1: At least a link to the nomenclature/symbols used for the monosaccharide units should be given. A key with the Figure would be helpful for non-glycobiologist.

Answer: Figure 1 has been modified accordingly. The identity of the employed monosaccharide symbols has been included, as suggested.

2) Figures 2 and S3: For the non-specialist in protein-NMR it would be nice to have an assignment of the different signal groups as aliphatic, aromatic etc. in Fig. S3. Looking at Figure 2 very critically, one identifies an additional signal in the LDN6 bound species at a ratio of 1:34. Of all spectra shown, it is only seen in this one spectrum. Any hint where this comes from? Wouldn't it make sense to also quote Figs. S8 and S12 in the text referring to Fig. 2?

   Answers: Thank you for the suggestion. A specific label detailing the region of the spectra (aliphatic/aromatic) has been added to Figures 2, S3, S8 and S12.

   Regarding the additional signal appearing in Figure 2 at a ratio of 1:34 of LDN6, we can only claim that it is an impurity. Actually we have realized that it is also present in the spectrum at high ratios (1:20) protein:LDN3, and have modified the figure accordingly. If we want to speculate, we think it could be silicone grease.

   We have also quoted Figures S8 and S12 in the main text, when mentioning Figure 2 and S3.

3) p. 4 and Figure S5: This is an interesting finding. Exchange is slow on the chemical shift time scale and at the same time saturation transfer is observed due to slow exchange on the relaxation time scale of the free ligand. Does this information maybe enable the authors to estimate an upper limit for the off-rate constant?

Answer: Yes, we have made an estimation of the k_{off} from the chemical exchange crosspeaks for the G0 oligosaccharide and for the disaccharide in the bound state. The EXSY analysis yielded an estimation of k_{off} = 0.2 s^{-1} and 1 s^{-1} for G0 and the disaccharide, respectively. We have included this analysis in the supporting information.

4) Figure 3: Please mention the saturation time and the number of scans (experiment time) in the legend. The nomenclature is not clear. In the spectra Man residues are labeled "C", "D", "E" whereas the cartoon to the right has "C", "3", "6". Maybe I have been missing the key to this, but I couldn't find it.

Answer: We thank the reviewer for pointing out this error. The nomenclature in Figure 3 was inaccurate and we have corrected it. We have also included the saturation time and number of scans for the legend of Figures 3 and 4.

5) p. 6: "Interestingly, the STD-NMR signals for the acetyl groups were always remarkably stronger under aliphatic irradiation, suggesting that the methyl groups are close to aliphatic residues of the protein." Can direct irradiation be excluded?

Answer: Yes. On resonance aliphatic frequency was set at δ 0.6ppm, 1.3ppm far from the signals of the acetyl groups, which is usually a very safe distance. Direct ligand irradiation using a 50 ms Gaussian shaped pulse (that results in narrow Gaussian excitation profile) would affect all the signals close to the irradiation frequency, providing larger intensities for those signals that are closest to the irradiation frequency and diminishing as we move towards lower field. For instance, for LDN6, signals corresponding to Ac-B and Ac-6 would be much more intense than those for Ac-3. This is not the case here where, in all cases, the Ac signals showing the strongest STD effect are the ones in the middle among the five Ac signals. Therefore, we can safely exclude direct irradiation.

We should additionally comment on the fact that we have employed very long relaxation delays (d1=15s), since we have experienced that the acetyl groups in these complex ligands relax very slowly and could give rise to false STD NMR signals. Thus, before setting the experiment, we estimated the T1 relaxation times for these signals and placed the d1 value accordingly.

6) Figure 6: Why are the two arms of different color?

Answer: We decided to represent the two arms of the G0 glycan with different colors to highlight the results (Figure 3): the STD NMR intensity observed for the

acetyl GlcNAc at the 3-branch displays double intensity than the acetyl group of the 6-branch GlcNAc. We have included a sentence in the figure caption to explain this fact.

7) MD simulations: It would be nice to have some details. Or did I misunderstand it and the discussion refers to published data only?

Answer: Yes. The discussion refers to the simulations performed specifically for this study. In order to provide more insights from the MD simulations and achieve better conformational sampling, we have run more independent replicas of model bCell-PEG8-G0 (28 independent 60 ns MD simulations, amounting to nearly 1.7 accumulated µs). We chose to run many replicas of short simulations instead of long ones for two reasons: 1) the large size of the solvated system, which would require very long wall-clock times. 2) the observed tendency of the glycan to quickly and irreversibly interact with the polar surface, particularly when short linkers are used. Thus, we decided to explore the intrinsic flexibility of the α(1→3) and α(1→6) linkages and their relative propensity to interact with the surface at short simulation times, while simultaneously exploring a much wider conformational space.

From these new simulations, we have also computed the Solvent-Accessible surface Area (SASA) for each terminal GlcNAc unit. Hence, we now provide statistically meaningful differences between the propensity of these two carbohydrates to interact with the polar surface.

This information has been added to the Supporting Information, and the discussion in the main text has been adapted and corrected accordingly.

Reviewer 2

Recommendation: Publish in ACS Central Science after minor revisions noted.

Comments:
Glycan array screening as become the dominant method for determining glycan-protein specificity. However, as this paper clearly demonstrates, immobilizing the glycan can alter the ability of the glycan to bind to a given protein. This is a very often over-looked issue, and this paper provides a compelling example of this phenomenon.

There are a few issues that should however be addressed that would push the technical quality into the top 1%.

1) The authors should confirm that the printed glycan in the array is not mis-printed or otherwise incorrect. This may be confirmed by demonstrating that the LDN3 ligand on the array binds as expected to other proteins. Array printing errors and other issues could otherwise explain the observation.

   Answer: The glycan array data to which the article refers was published previously in ACS *Chem. Biol.* 2018, 13, 8, 2269–2279 (reference 22 in the manuscript). In this paper, binding data for glycan arrays with three anti-LeX monoclonal IgM antibodies and the 3 immune lectins: DC-SIGN, L-sectin, and DC-SIGNR were reported. Branch dependent binding of isomers was also observed for DC-SIGN and DC-SIGNR (with inverse selectivity for DC-SIGN and DC-SIGNR) clearly demonstrating that glycans had been printed correctly. DC-SIGN bound one isomer but not the other, while DC-SIGNR bound the isomer that DC-SIGN did not bind to and vice versa.) In addition, L-sectin branch specificity was found for 3 different isomers: with terminal GalNAc (as in this manuscript), terminal galactose and a LDNF structural element on the 6-arm.
   Microarray printing is performed with a piezoelectric non-contact printer that contains certain controls such as pre-drop and post-drop dispensing by image, a final report with the missing spots (if any) and the final images of all drops printed, where misprinted slides could also be ruled out. Furthermore, after each microarray printing run of typically 25 slides, one microarray slide is always checked with control plant lectins to ensure the correct immobilization on the microarrays. The image below shows the identical recognition of both isomeric N-glycans by three different lectins: *Wisteria floribunda agglutinin* (WFL) and *Erythrina crystagalli agglutinin* (ECA) both selective for Gal/GalNAc, and *Banderia simplicifolia* lectin (BSL II) selective for terminal GlcNAc. These data are coming from the printing control incubation of the data included in the publication ACS Chem. Biol. 2018, 13, 8, 2269–2279 and thus, we can safely consider that the microarrays are correctly printed.

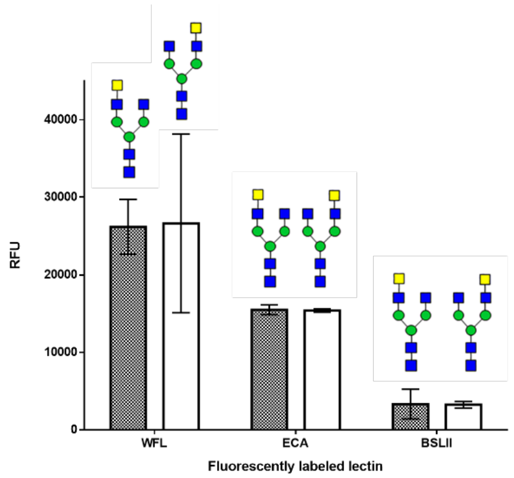

2) The authors illustrate with a figure the accessibility of the 1-3 arm of the glycan in the MD simulation snapshot. However, they need to quantify this accessibility over the course of the simulation, and similarly quantify the level of accessibility for the 1-6 arm.

   Answer: Yes. We thank the reviewer for his/her useful suggestion. In order to provide more insights from the simulations and achieve better conformational sampling, we have run many more independent replicas of model bCell-PEG8-G0 (28 independent 60 ns MD simulations, amounting to nearly 1.7 accumulated μs). We chose to run many replicas of short simulations instead of long ones for two reasons: 1) the large size of the solvated system, which would require very long wall-clock times, and 2) the observed tendency of the glycan to quickly and irreversibly interact with the polar surface, particularly when short linkers are used. Thus, we decided to explore the intrinsic flexibility of the α(1→3) and α(1→6) linkages and their relative propensity to interact with the surface at short simulation times while simultaneously exploring a much wider conformational space.
   Following the reviewer's recommendation, we have also computed the Solvent-Accessible surface Area (SASA) for each terminal GlcNAc unit along these new simulations. Hence, we now provide statistically meaningful differences between the propensity of these two carbohydrates to interact with the polar surface in the form of histograms of the SASA values derived from the accumulated trajectories.
   This information has been added to the Supporting Information, and the discussion in the main text has been adapted and corrected accordingly.

3) I urge the authors not to refer to G0 as a symmetric glycan. Because the core contain 1-3 and 1-6 linkages, it is NOT symmetric and this word choice rather goes against their efforts to present the idea that these arms are not actually equivalent. This is an important point because the use of the SNFG symbols tends to further imply symmetry.

Answer: Yes, we completely agree, thank you for this observation. We have made the appropriate changes and refer to G0 as the "non-elongated N-glycan".
